# Supplementary material for: E-cigarette adverts and children’s perceptions of tobacco smoking harms: an experimental study and meta-analysis
Source: BMJ Open. 2018 Jul 16;8(7):e020247. doi: 10.1136/bmjopen-2017-020247 (PMC6082488; doi:10.1136/bmjopen-2017-020247)
Supplement: Supplementary file 1 [file bmjopen-2017-020247supp001.pdf]

ONLINE SUPPLEMENTARY MATERIALS:

E-CIGARETTE ADVERTS AND CHILDREN'S PERCEPTIONS OF TOBACCO SMOKING HARMS: AN EXPERIMENTAL STUDY AND META-ANALYSIS

Milica Vasiljevic[a], PhD, Amelia St John Wallis[a], BA, Saphsa Codling[a], MA, Dominique-Laurent Couturier[a], PhD, Stephen Sutton[a,b], PhD, & Theresa M. Marteau[a], PhD

[a] Behaviour and Health Research Unit, Institute of Public Health, University of Cambridge, Cambridge, UK

[b] Behavioural Science Group, Institute of Public Health, University of Cambridge, Cambridge, UK

Appendix A: Exploratory Analyses .... pp. 2-6

Appendix B: Details of searches for meta-analysis .... pp. 7-11

Appendix A: Exploratory Analyses

Exploratory Analyses

We carried out two sets of exploratory analyses. First, multiple ordinal regressions (non-parametric) were conducted to examine any potential interactions between the experimental groups and age, gender, or ethnicity on the primary outcome (perceived harm of occasional tobacco smoking). The results of these ordinal regressions are reported in Tables S1 to S3 below. None of these three demographic variables moderated the effect of experimental group on perceived harm of occasional tobacco smoking (*ps* > .05).

Second, we carried out exploratory analyses on the subsample of ever-smokers and ever-users of e-cigarettes (*n* = 362). We repeated all analyses carried out on the sample of non-smokers and non-e-cigarette users also in this subsample of ever-smokers and ever-users of e-cigarettes.

The only significant differences between the experimental conditions were on the indices of appeal (*p* = .037), and interest in buying and trying the products shown in the adverts (*p* = .032). No other effects reached the threshold of significance (see Table S4 below). These analyses should be considered with caution, since they are exploratory, and likely statistically underpowered given the sample size of ever smokers and e-cigarettes users is very small.

Table S1.

Exploratory analyses of the interaction between experimental group and age on perceived harm of occasional tobacco smoking

**Variable**	**Estimate**	**S.E.**	***Z***	***p*-value**
Experimental Group	-0.21049	0.11536	-1.825	0.0681
Age	0.19659	0.08389	2.343	0.0191*
ExpGroup X Age	-0.19647	0.11555	-1.700	0.0891

Note. *denotes significance at <.05.

Table S2.

Exploratory analyses of the interaction between experimental group and gender on perceived harm of occasional tobacco smoking

**Variable**	**Estimate**	**S.E.**	***Z***	***p*-value**
Experimental Group	-0.13846	0.16358	-0.846	0.397
Gender	-0.09726	0.16356	-0.595	0.552
ExpGroup X Gender	-0.14149	0.23077	-0.613	0.540

Table S3.

Exploratory analyses of the interaction between experimental group and ethnicity on perceived harm of occasional tobacco smoking

Variable	Estimate	S.E.	*Z*	***p*-value**
Experimental Group	-0.28055	0.13027	-2.154	0.0313*
Ethnicity	-0.09271	0.20358	-0.455	0.6488
ExpGroup X Ethnicity	0.24586	0.28323	0.868	0.3854

Note. *denotes significance at <.05.

Table S4.

Descriptive statistics across experimental groups for ever-smokers and ever-users of e-cigarettes (Exploratory analyses)

Outcome Variable	E-cig Adverts (*n* = 175)	Control Adverts (*n* = 187)	Test statistic	*p*-value
Perceived harm of occasional tobacco smoking	179.02	183.82	-.455	.649
Perceived harm of tobacco smoking in general	183.53	179.60	-.419	.675
Perceived harm of regular tobacco smoking	180.71	182.24	-.154	.878
Perceived disease risk (regular smoking)	182.85	180.24	-.240	.810
Perceived disease risk (occasional smoking)	177.62	183.23	-.516	.606
Tobacco smoking prevalence estimates	180.32	177.76	-.234	.815
Susceptibility to tobacco smoking	81.7	82.4	.376	.540
Perceived harm of occasional e-cigarette use	177.51	184.28	-.748	.454
Perceived harm of e-cigarette use in general	176.83	184.92	-.779	.436
Perceived harm of regular e-cigarette use	174.89	186.74	-1.125	.261
Perceived disease risk (regular e-cig use)	176.22	183.55	-.680	.496
Perceived disease risk (occasional e-cig use)	176.11	182.70	-.680	.497
E-cigarette use prevalence estimates	179.77	178.28	-.136	.892
Susceptibility to e-cig use	94.9	95.7	.022	.883
Appeal of adverts	169.65	192.59	-2.088	.037
Interest in buying and trying advertised product	169.33	192.89	-2.144	.032

Note: For all outcome variables the test statistic corresponds to the *Z* value from the Mann Whitney *U* analyses (with corresponding Mean Ranks shown for each experimental group), except for the variables susceptibility to smoking and e-cigarettes use which are binary variables and are denoted by percentages summarised using the X^2 test statistic.

Appendix B: Details of searches for meta-analysis

Eligibility criteria

Only randomised studies with any length of follow-up were included if they assessed exposure to e-cigarette adverts of any nature amongst children and adolescents. Eligible comparators were: (a) exposure to non-e-cigarette adverts; or (b) no exposure to adverts. Eligible studies also had to assess the effects of exposure in terms of the following outcome: perceived harm of occasional tobacco smoking. Studies that used non-randomised designs were not eligible. Studies that did not examine the effect of e-cigarette advertisements were also ineligible. Only studies reported in English were considered eligible. There were no eligibility restrictions for study publication status or date.

Search methods and study selection procedures

Eligible studies were located using electronic searches of PubMed and Google Scholar™. Keywords used in the database searches were combinations of the terms: ‘e-cigarette adverts’ (OR ‘e-cigarette advertisements, OR ‘e-cigarette marketing’, OR ‘electronic cigarette adverts’, OR ‘electronic cigarette advertisements, OR ‘ electronic cigarette marketing’), AND ‘children’ (OR ‘adolescents’), AND ‘perceived harm of occasional tobacco smoking’ (OR ‘harm of tobacco smoking’, OR ‘perceived harm of tobacco smoking’). Searches were conducted between 5 June and 17 July 2017 (and repeated between 25 January and 11 February 2018). Provisional eligibility decisions based on title-abstract screening were made by one reviewer (MV). Final eligibility decisions, based on examination of full-text study reports, were made by one reviewer (MV) and checked by a second (ASJW).

Data collection, risk of bias assessment and analysis

Data on the characteristics and results of included studies were extracted by one reviewer (MV) and checked by a second (ASJW). Study-level effect sizes were computed for the eligible outcome measure as the standardised mean difference (SMD) between comparison groups.

Study-level effect sizes were next combined using a fixed-effects meta-analysis due to the small sample size of identified eligible studies (k = 3), conducted using Review Manager 5.3. Statistical heterogeneity was assessed by inspection of graphical displays of each SMD and its 95% confidence interval, and a formal statistical test of homogeneity (I^2).

Results of the search

Bibliographic details of all studies identified by searches in PubMed and Google Scholar™ are provided below. Both sources yielded a total of eight primary study records (and one literature review). The eight primary study records were screened. Six primary studies were excluded based on screening, due to the studies not using randomised designs. Two studies were accepted for the meta-analysis (Petrescu et al., 2017; Vasiljevic et al., 2016), and were synthesised together with the primary data reported in the present manuscript (Vasiljevic et al., 2018). For characteristics of all three included studies in the meta-analysis see Table S5 below.

Table S5. Characteristics and results of included randomised controlled trials (*k* = 3).

Study	*Funding source*	*Design*	*Country, setting*	*Participants that completed study*	*Intervention(s)*	*Comparator(s)*	*Outcome measure (Perceived harm of occasional tobacco smoking)*	*Effect of exposure to e-cigarette adverts*	*Study-level effect size (SMD and 95% CI)*
Petrescu et al., 2017	Department of Health Policy Research Programme (Policy Research Unit in Behaviour and Health [PR-UN-0409-10109]).	RCT	UK, Home setting.	411 school children aged 11-16 years (M=13.09yrs, SD=1.68); 52.8% female.	Exposure to glamorous e-cigarette adverts; OR health-related e-cigarette adverts.	No e-cigarette adverts shown.	Single item rated on a five point scale, 1 = Not very dangerous to 5 = Very dangerous..	↓	-0.33 (-0.54 to -0.13)
Vasiljevic et al., 2016	Department of Health Policy Research Programme (Policy Research Unit in Behaviour and Health [PR-UN-0409-10109]).	RCT	UK, School setting.	471 school children aged 11-16 years (M=13.06yrs, SD=1.48); 48.2% female.	Exposure to candy flavoured e-cigarette adverts; OR non-candy flavoured e-cigarette adverts.	No e-cigarette adverts shown.	Single item rated on a five point scale, 1 = Not very dangerous to 5 = Very dangerous..	→←	-0.07 (-0.26 to 0.12)

Vasiljevic et al., 2018	Department of Health Policy Research Programme (Policy Research Unit in Behaviour and Health [PR-UN-0409-10109]).	RCT	UK, School setting.	1057 school children aged 11-16 years (M=13.48yrs, SD=1.37); 53.1% female.	Exposure to glamorous e-cigarette adverts. .	Exposure to pen adverts.	Single item rated on a five point scale, 1 = Not very dangerous to 5 = Very dangerous..	↓	-0.12 (-0.24 to 0.00)

Bibliographies of identified studies

Included studies

Petrescu et al., 2017

Petrescu DC, Vasiljevic M, Pepper JK, et al. What is the impact of e-cigarette adverts on children's perceptions of tobacco smoking? An experimental study. *Tob Control* 2017;26:421-27.

Vasiljevic et al., 2016

Vasiljevic M, Petrescu DC, Marteau TM. Impact of advertisements promoting candy-like flavoured e-cigarettes on appeal of tobacco smoking among children: an experimental study. *Tob Control* 2016;25(e2):e107-e12.

Vasiljevic et al., 2018

Vasiljevic M, St John Wallis A , Codling S, Couturier D-L, Sutton S, Marteau TM. E-cigarette adverts and children's perceptions of tobacco smoking harms: An experimental study. *BMJ Open* (under review).

Excluded study reports

Bauld L, Angus K, Ford A. *Electronic Cigarette Marketing: Current Research and Policy.* Cancer Research UK. 2016.

Conner M, Grogan S, Simms-Ellis R, Flett K, Sykes-Muskett B, Cowap L, Lawton R, Armitage CJ, Meads D, Torgerson C, West R. Do electronic cigarettes increase cigarette smoking in UK adolescents? Evidence from a 12-month prospective study. *Tob Control* 2017 doi:10.1136/tobaccocontrol-2016-053539.

Ford A, MacKintosh AM, Bauld L, Moodie C, Hastings G. Adolescents' responses to the promotion and flavouring of e-cigarettes. *Int J Public Health* 2016;61:215-24.

McKeganey N, Barnard M, Russell C. Visible vaping: E-cigarettes and the further de-normalization of smoking. *Int Arch Addict Res Med* 2016;2:1-6.

Pasch KE, Nicksic NE, Opara SC, Jackson C, Harrell MB, Perry CL. Recall of Point-of-Sale Marketing Predicts Cigar and E-Cigarette Use among Texas Youth. *Nicotine Tob Res* 2017 doi:10.1093/ntr/ntx237.

Popova L, So J, Sangalang A, Neilands TB, Ling PM. Do Emotions Spark Interest in Alternative Tobacco Products?. *Health Educ Behav* 2017;44:598-612.

Reinhold B, Fischbein R, Bhamidipalli SS, Bryant J, Kenne DR. Associations of attitudes towards electronic cigarettes with advertisement exposure and social determinants: a cross sectional study. *Tob Induc Dis* 2017;15:13.
