# Supplementary material for: E-cigarette adverts and children’s perceptions of tobacco smoking harms: an experimental study and meta-analysis
Source: BMJ Open. 2018 Jul 16;8(7):e020247. doi: 10.1136/bmjopen-2017-020247 (PMC6082488; doi:10.1136/bmjopen-2017-020247)
Supplement: Author's manuscript [file bmjopen-2017-020247.draft_revisions.pdf]

BMJ Open

BMJ Open is committed to open peer review. As part of this commitment we make the peer review history of every article we publish publicly available.

When an article is published we post the peer reviewers' comments and the authors' responses online. We also post the versions of the paper that were used during peer review. These are the versions that the peer review comments apply to.

The versions of the paper that follow are the versions that were submitted during the peer review process. They are not the versions of record or the final published versions. They should not be cited or distributed as the published version of this manuscript.

BMJ Open is an open access journal and the full, final, typeset and author-corrected version of record of the manuscript is available on our site with no access controls, subscription charges or pay-per-view fees (http://bmjopen.bmj.com).

If you have any questions on BMJ Open's open peer review process please email info.bmjopen@bmj.com

BMJ Open

E-cigarette adverts and children's perceptions of tobacco smoking harms: An experimental study

Journal:	*BMJ Open*
Manuscript ID	bmjopen-2017-020247
Article Type:	Research
Date Submitted by the Author:	23-Oct-2017
Complete List of Authors:	Vasiljevic, Milica; Behaviour and Health Research Unit<br>St John Wallis, Amelia; Behaviour and Health Research Unit<br>Codling, Saphsa; Behaviour and Health Research Unit<br>Couturier, Dominique-Laurent; Behaviour and Health Research Unit<br>Sutton, Stephen; University of Cambridge,<br>Marteau, Theresa; University of Cambridge, Behaviour and Health Research Unit
**Primary Subject Heading**:	Smoking and tobacco
Secondary Subject Heading:	Communication, Public health
Keywords:	PREVENTIVE MEDICINE, PUBLIC HEALTH, STATISTICS & RESEARCH METHODS

SCHOLARONE™
Manuscripts

For peer review only

E-CIGARETTE ADVERTS AND CHILDREN'S PERCEPTIONS OF TOBACCO SMOKING HARMS: AN EXPERIMENTAL STUDY

Milica Vasiljevic[a], PhD, Amelia St John Wallis[a], BA, Saphsa Codling[a], MA, Dominique-Laurent Couturier[a], PhD, Stephen Sutton[a,b], PhD, & Theresa M. Marteau[a], PhD

Affiliations: [a] Behaviour and Health Research Unit, Institute of Public Health, University of Cambridge, Cambridge, UK; and [b] Behavioural Science Group, Institute of Public Health, University of Cambridge, Cambridge, UK

Address correspondence to: Milica Vasiljevic, Behaviour and Health Research Unit, Institute of Public Health, Addenbrooke's Hospital, University of Cambridge, Cambridge, CB2 0SR, UK [milica.vasiljevic@medschl.cam.ac.uk], tel: +44 (0) 1223 761314.

Word Count: 3,404 (text only)

Number of Tables: 3

Number of Figures: 1

Abbreviations:

E-cig – e-cigarette

Advert – advertisement

For peer review only

ABSTRACT

Objectives: Children exposed to e-cigarette adverts may perceive occasional tobacco smoking as less harmful than children not exposed to e-cigarette adverts (Petrescu et al., 2016).[1] Given the potential cross-cueing effects of e-cigarette adverts on tobacco smoking there is an urgent need to establish whether the effect found in prior research is robust and replicable using a larger sample and a stronger control condition.

Design: A between-subjects experiment with one independent factor of two levels corresponding to the advertisements to which participants were exposed: glamorous adverts for e-cigarettes, or adverts for objects unrelated to smoking or vaping.

Participants: English school children aged 11-16 (*n*=1449).

Outcomes: Perceived harm of occasional smoking of one or two tobacco cigarettes was the primary endpoint. Secondary endpoints included: perceived harm of regular tobacco smoking, susceptibility to tobacco smoking and perceived prevalence of tobacco smoking in young people. All endpoints were adapted to assess perceptions of using e-cigarettes.

Results: Tobacco smokers and e-cigarette users were excluded from analyses (final sample *n*=1057). Children exposed to glamorous e-cigarette adverts perceived the harms of occasional smoking of one or two tobacco cigarettes to be lower than those in the control group (Z=-2.13, p=.033). An updated meta-analysis comprising three studies with 1935 children confirmed that exposure to different types of e-cigarette adverts [glamorous, healthful, flavoured, non-flavoured] lowers the perceived harm of occasional smoking of one or two tobacco cigarettes (Z=3.21, p=.001).

Conclusions: This study adds to existing evidence that exposure to e-cigarette adverts reduces children's perceptions of the harm of occasional tobacco smoking.

Keywords: advertising and promotion; electronic nicotine delivery devices; priority/special populations; non-cigarette tobacco products

For peer review only

STRENGTHS AND LIMITATIONS OF THIS STUDY:

- We replicate and extend prior findings regarding the perceived harm of occasional tobacco smoking using a larger sample and a stronger control condition.
- Meta-analysis of three studies confirms that exposing children to different e-cigarette adverts [glamorous, healthful, flavoured, or non-flavoured] lowers their perceived harm of occasional tobacco smoking.
- The present study was limited in two respects: the primary outcome measured perceived risk of smoking, not behaviour; and the design used only momentary exposure to e-cigarette adverts.
- Future studies should examine whether perceptions of harm following exposure to e-cigarettes translates into less negative attitudes towards tobacco smoking, the tobacco industry and in turn tobacco control policies.
- Future studies should also use longitudinal experimental designs with behavioural outcomes to corroborate the present findings.

For peer review only

INTRODUCTION

Fewer children are smoking tobacco cigarettes today than several decades ago. However, the advent of electronic cigarettes (e-cigarettes) could disrupt this trend. The availability and use of e-cigarettes has risen rapidly in the last four years with an estimated 12%-24% of children aged 11-18 experimenting with e-cigarettes in the UK in 2015/16,[2] and 5.3% of middle schoolers and 16% of high schoolers in the USA in 2015.[3,4]

E-cigarettes have the potential for benefit and harm, the nature and scale of each being uncertain in the absence of much evidence. One potential benefit comes from providing a safe delivery mechanism for nicotine and an effective cessation aid. Evidence is accumulating to suggest that e-cigarettes can successfully be used as cessation aids by smokers.[5,6] Of concern, however, is their potential to make attitudes towards tobacco smoking more positive (i.e., to renormalise it) through, for example, marketing of objects that appear very similar to tobacco cigarettes that appeal to both adult and children non-smokers. Any such impact on children is of particular concern given the potential for any changes in attitudes to tobacco smoking to increase the chances of tobacco smoking in this group in particular. [4,7,8]

Several prospective studies in the USA and UK have found that among children e-cigarette use predicts tobacco smoking one year later.[9-13] By contrast, population level data show that the rising use and experimentation of e-cigarettes among children has been accompanied by a continued decline in tobacco smoking in that group from 15.8% to 9.2% amongst US high-schoolers in the period from 2011 to 2014,[3] and from 5% in 2010 to 3% in 2014 amongst 11-15

For peer review only

year olds in England.[14] Any impact on tobacco use of the recent upsurge in e-cigarette use in children will become more certain as the period of observation is extended. Experimental studies can also provide pertinent evidence.

The limited experimental evidence concerning the impact of e-cigarette exposure on children has focused on exposure to e-cigarette advertising. In one study, children exposed to televised e-cigarette adverts expressed more positive attitudes towards and greater intentions to use e-cigarettes.[15] In another study, children seeing candy flavoured e-cigarette adverts found these adverts more appealing and were more interested in buying and trying the products when compared to those children exposed to non-flavoured e-cigarette adverts.[16] But in neither study did exposure to e-cigarette advertisements significantly increase the appeal of smoking tobacco cigarettes. Only one study to date has found a cross-product influence of e-cigarette adverts on perceptions of the harms of occasional tobacco smoking.[1] In this study, exposing children to e-cigarette adverts characterised as depicting glamour or health had no significant impact on the appeal of smoking tobacco cigarettes, or the perceived harm of smoking more than 10 cigarettes per day. However, those exposed to either set of adverts perceived the harms of smoking one or two tobacco cigarettes occasionally to be lower than did those not exposed to any adverts.

Even though the size of the effect of perceived risk on routine or habitual behaviours is small to moderate,[17,18] it is nonetheless important in this context given the harms of tobacco smoking. Perceived harm of occasional smoking predicts tobacco smoking.[19,20] Furthermore, although the health consequences of occasional smoking can be as severe as regular smoking,[21] young

For peer review only

smokers who smoke occasionally do not consider themselves smokers, believing they are immune to the risks associated with smoking, and have low intentions to quit.[22,23] In a similar vein, perceived risk significantly predicts intentions and behaviours generally,[17,18] as well as more specifically in relation to smoking, with perceived harm associated with greater likelihood of staying abstinent or quitting if smoker.[24-26]

The aim of the present study is to replicate and extend recent findings showing that children perceive the harms of occasional tobacco smoking to be lower after exposure to e-cigarette adverts. By using a larger sample of children aged 11-16 and a control condition with equivalent task demands in which children were exposed to adverts for objects unrelated to tobacco smoking or vaping (pens), we sought to provide a more robust estimate of the effect found by Petrescu and colleagues.[1]

METHODS

Design

A between-subjects experiment with one independent factor of two levels corresponding to the advertisements to which participants were exposed:

A. Adverts depicting e-cigarette use as glamorous

B. Adverts for objects [pens] unrelated to tobacco smoking or vaping (control condition)

Participants

For peer review only

Data were collected from 1449 English school children aged between 11-16 years (sampled from three schools, two based in Cambridgeshire and one based in Hampshire). Data were collected and analysed in 2016. Randomisation was successful: there were no significant differences between the two experimental groups on any of the demographic, smoking or e-cigarette use characteristics measured. Ever-smokers and ever-users of e-cigarettes were excluded from the analyses leaving a final sample of 1057 participants. Characteristics of the full and final samples are shown in Table 1a and 1b respectively. This sample size provided more than 90% power at $\alpha = .05$ to detect a small-sized effect of glamorous e-cigarette adverts upon the perceived harm of occasional tobacco smoking (based on a recent study by Petrescu et al),[1] allowing for a reduction in sample size caused by excluding children with prior tobacco smoking or e-cigarette use.[27]

====================== **PLACE TABLE 1 HERE** ======================

Intervention

Each experimental condition displayed 10 adverts, with the control condition showing adverts of pens, and the e-cigarette condition showing adverts associating e-cigarette use with glamour. The e-cigarette adverts were taken from Petrescu et al.[1] The e-cigarette adverts for that study were sampled from the Stanford Adverts Repository (http://tobacco.stanford.edu/tobacco_main/index.php). A subset of 40 possible e-cigarette adverts were pilot tested with 16 year olds. Ten adverts were selected based on ratings for their depiction of glamour (for more details see Petrescu et al).[1] The adverts for the control condition were selected from a larger sample of pen adverts. The pen adverts were sourced online. Pen adverts were chosen as the control stimuli due to their similar shape and look to tobacco and e-cigarettes. Three authors (MV, ASJW, SC) selected pen adverts to match the content of the e-cigarette

For peer review only

adverts, including the presence of a person (with four adverts showing a woman using a pen, four adverts showing a man using a pen, and two adverts with no person in the advert).

Measures

Primary endpoint:

Perceived harm of occasional tobacco smoking was assessed by an item adapted from Wakefield et al.[28] *"How dangerous do you think it is to smoke one or two cigarettes occasionally?"* rated on a five point scale, *1 = Not very dangerous* to *5 = Very dangerous*.

Secondary endpoints:

Perceived harm of tobacco smoking regularly and in general was measured using two items.[28] "*Smoking can harm your health"* rated from *1 = Strongly disagree* to *5 = Strongly agree*, and *"How dangerous do you think it is to smoke more than 10 cigarettes a day?"* rated from *1 = Not very dangerous* to *5 = Very dangerous*. These were analysed separately as in previous studies.[28,29]

Perceived risk of developing tobacco related diseases was measured by items adapted from Pepper et al.[30] *"How likely do you think it is that smoking tobacco cigarettes more than 10 times a day regularly [smoking tobacco cigarettes once or twice occasionally] would cause you to develop each of the following in the next 10 years? (If you're not sure, please give us your best*

For peer review only

guess) (a) lung cancer, (b) heart disease and (c) mouth or throat cancer" Ratings were provided on scales from *1= Not at all likely* to *5 = Extremely likely.* Two separate composite indices were made for perceived risk from regular (α=.76) and occasional (α=.90) tobacco smoking respectively.

Prevalence estimates of tobacco smoking were given on an open-ended question: "*How many young people your age out of 100 do you think smoke tobacco cigarettes?"*.[31]

Susceptibility to tobacco smoking was measured using three items: *"Do you think you will be smoking tobacco cigarettes when you are 18 years old?"*; *"Do you think you will smoke a tobacco cigarette at any time during the next year?"* and *"If one of your friends offered you a tobacco cigarette, would you smoke it?"*.[32] Participants were categorised as susceptible if they did not respond *"definitely not"* to all three items.

Appeal of adverts was assessed by asking: *"How much do you like this advert (not the product)?"*.[33] Responses ranged from *1 = Not at all* to *4 = A lot*. Responses to the adverts were averaged into a single index (α=.80).

Interest in buying and trying products displayed in the adverts was assessed with the item: *"Does this advert make you want to buy and try this product?"* with scores ranging from *1 = Not at all* to *4 = Yes, a lot.*[33] Responses were averaged across the 10 adverts (α=.85).

For peer review only

E-cigarette use related outcomes: All endpoints were adapted to also assess perceptions of using e-cigarettes. The composite indices for perceived risk from regular (α=.93) and occasional (α=.95) e-cigarette use had good inter-item reliabilities.

Other measures

Tobacco smoking was measured with two items: *"Have you ever smoked a tobacco cigarette?"* and *"Have you ever tried tobacco cigarette smoking, even one or two puffs?".*[32] Items assessing tobacco cigarette smoking were adapted to assess *use of e-cigarettes*: *"Have you ever used an e-cigarette?"* and *"In the past 30 days, on how many days did you use an e-cigarette?"* For dual users we also asked: *"If you are both smoking tobacco cigarettes and using e-cigarettes, which product did you start using first?"* Gender, age, and ethnicity were also recorded.

Procedure

University of Cambridge's Psychology Research Ethics Committee approved the study [PRE.2015.106]. Prior passive parental consent was obtained, and the head-teachers of the schools acted *in-loco parentis* during data collection. Before commencing the study participating children were reminded that they could withdraw from the study at any point.

The study materials were presented in paper-pencil format, with each participant receiving a booklet corresponding to one of the two experimental conditions depending on randomisation. Participants in the e-cigarette and control adverts conditions were each exposed to a series of 10

For peer review only - http://bmjopen.bmj.com/site/about/guidelines.xhtml

print-adverts in their booklets. To ensure that participants engaged with the adverts, after each advert they were asked to rate the appeal of the advert, and their interest in buying and trying the product (see Measures). Children in both experimental conditions were told the study was about their views on e-cigarettes and tobacco cigarettes. Children completed the experimental booklets at their own pace, and exposure to the adverts was not timed. The order in which the adverts appeared was fixed across participants. Potential confusion between e-cigarettes and tobacco cigarettes was managed by: (a) presenting all items pertaining to tobacco cigarettes and e-cigarettes in two separate sections; (b) adding a heading at the beginning of each section informing participants that the next section will deal with either tobacco or e-cigarettes; (c) including a picture of a tobacco cigarette and a picture of an e-cigarette at the beginning of each section; and (d) including a definition of e-cigarettes before the presentation of adverts and before assessing e-cigarette related items.

Participants were randomly assigned to one of the two groups, using a pre-established random sequence generated by the statistical package R. Prior to the testing session the different versions of the booklets were arranged in the pre-randomised order and these booklets were then distributed during testing. Experimenters made sure that participants finishing earlier than others remained seated until everyone had finished. Once participants had completed their questionnaires, they were provided with a verbal and written debrief about the nature of the study.

Statistical Analysis

All analyses were conducted using SPSS (version 23) and R (version 3.3.1). Responses on the primary and secondary outcomes were not normally distributed. Subsequent analyses were

For peer review only

therefore conducted using non-parametric statistical tests (Mann-Whitney *U* and ordinal regression) to test equality of the location parameter between treatment groups. To provide a summary of the effects of e-cigarette advertising on perceived harm of tobacco smoking, we meta-analysed the present data and the results of two published studies that also examined the impact of different types of e-cigarette adverts on perceptions of tobacco harm.[34] We searched published records for studies that could be synthesised, so the meta-analysis provides an accurate representation of all evidence currently available to us. All measures, experimental conditions, and sample size calculations are reported in the manuscript. For descriptive statistics see Tables 2 and 3.

====================== PLACE TABLES 2 & 3 HERE ======================

RESULTS

Primary endpoint

Perceived harm of occasional tobacco smoking: Children exposed to glamorous e-cigarette adverts (Mean Rank = 508.69) perceived the danger as lower than did the control group (Mean Rank = 546.84), (Mann-Whitney U = 129045.500, Z = -2.129, p = .033). Using ordinal regression (controlling for clustering at the level of school) replicated these results (t=-2.131, p = .033).

Secondary endpoints

There were no statistically significant differences between the groups in the perceived harm of regular smoking (or using e-cigarettes) and smoking (or using e-cigarettes) in general, perceived

For peer review only

risk of developing tobacco-related diseases due to regular and occasional smoking (or using e-cigarettes), perceived susceptibility to smoking tobacco cigarettes (or using e-cigarettes), or the prevalence estimates for tobacco smoking (or using e-cigarettes).

Children exposed to glamorous e-cigarette adverts (Mean Rank = 426.32) liked the adverts less than did those in the control group (Mean Rank = 628.80), (Mann-Whitney $U = 86133.500$, $Z = -10.797$, $p < .001$). Furthermore, children exposed to glamorous e-cigarette adverts (Mean Rank = 393.83) were less interested in buying and trying the products shown in the adverts than were those in the control group (Mean Rank = 660.39), (Mann-Whitney $U = 69202.500$, $Z = -14.298$, $p < .001$)

Meta-analysis

The same measure of perceived harm of occasional tobacco smoking was used in two other, similar studies. These assessed the impact of exposure to candy-like flavoured and non-flavoured e-cigarette adverts,[16] and the impact of glamorous and healthful e-cigarette adverts.[1] Using results from these two studies and the current study, we conducted a meta-analysis (using Review Manager version 5.3) of the continuous outcome, comparing those exposed to any type of advert for e-cigarettes with those in the control groups.

Exposing children to adverts for e-cigarettes decreases their perceived harm of occasional tobacco smoking: SMD = -0.15, 95% CI [-0.24, -0.06], $I^2 = 48\%$, Z = 3.21, $p = .001$ (see Figure 1). Similar results were obtained when dichotomising responses to this outcome (as in Petrescu et al.).[1]

For peer review only

======================= **PLACE FIGURE 1 HERE** ======================

DISCUSSION

Children exposed to e-cigarette adverts depicting glamour perceived the harms of smoking one or two tobacco cigarettes occasionally to be lower than did those exposed to unrelated adverts. These results corroborate previous findings.[1] An updated meta-analysis comprising three studies (including the present study) with 1935 children confirmed that exposure to different types of e-cigarette adverts [glamorous, healthful, flavoured, or non-flavoured] lowers the perceived harm of occasional smoking of one or two tobacco cigarettes. The current study also replicates previous findings that exposure to glamorous and other types of adverts does not affect children's perceptions of the (high) harm of regularly smoking more than 10 tobacco cigarettes per day.[1,16] Our findings suggest that exposure to adverts for e-cigarettes may lead to differences in how children perceive the harms of tobacco smoking.

The absence of a significant impact of viewing e-cigarette adverts upon perceptions of the harms associated with regularly smoking more than 10 tobacco cigarettes a day is encouraging [see also[1,16]]. However, the impact on perceived harms of occasional smoking is concerning given that such perceptions can predict subsequent smoking.[19,20] Young occasional smokers in particular do not consider themselves smokers, believing they are immune to the risks associated with smoking, with low intentions to quit.[22,23] The effect of e-cigarette adverts on perceived harms of occasional tobacco smoking is therefore both theoretically and empirically important, given that perceived harm (risk) is a key construct affecting health behaviour change in multiple theories of behaviour change [see[35]]. Furthermore, the observed differential effects on the perceived harms of occasional *vs.* regular smoking may provide an indication that the former

For peer review only - http://bmjopen.bmj.com/site/about/guidelines.xhtml

behaviour may be easier to mentally 'justify', thereby providing another potential route to self-regulation failure.[36]

In more general terms, the population consequences of our findings are currently unknown. Two sets of outcomes need to be considered. First, the possible impact on tobacco smoking and second the possible impact on attitudes towards tobacco smoking. First, a small change in perceived harm of occasional smoking and no change in the already high perceived harm of smoking 10 or more cigarettes on a regular basis, may have no impact on the likelihood that children smoke tobacco cigarettes. This is supported by the evidence that perceived harms of occasional tobacco smoking have a small to moderate effect on actual smoking.[19,20] It is also consistent with the evidence that despite exposure to adverts and vaping there is no corresponding increase in the overall rates of children smoking tobacco. Indeed, the decline in rates observed over the last two decades has continued.[27,37] Nonetheless, any impact of e-cigarette adverts on tobacco smoking in children demands attention from policy makers.

Second, a lower perceived harm of occasional smoking may lead to a less negative attitude towards tobacco smoking, the tobacco industry and in turn tobacco control policies. In high income countries public attitudes towards tobacco control policies, particularly those targeting children, are very positive.[38,39] Such attitudes are important in supporting policy makers in implementing effective tobacco control policies. Any lessening of these positive attitudes towards tobacco control would be a concern.

Strengths and Limitations with Future Directions

For peer review only

The large sample of children, and the use of a control condition in which children were exposed to a battery of adverts of objects unrelated to tobacco cigarettes or e-cigarettes strengthen the conclusions that can be drawn from the present study. By using a control condition in which children were exposed to pen adverts we were able to isolate the effects of e-cigarette adverts, and conclude that findings of lowered harm of occasional tobacco smoking can be attributed to e-cigarette adverts and not to viewing adverts more generally. Another strength of the current study is its contribution to an updated meta-analysis providing the most robust evidence to date that e-cigarette adverts of different kinds [glamorous, healthful, flavoured, or non-flavoured] may have a cross-product influence in lowering children's perceptions of the harms of occasional tobacco smoking.

The study was limited in two respects. The primary outcome was a belief and not a behaviour. Future studies should examine whether perceptions of harm following exposure to e-cigarettes translates into actual smoking behaviour.

The study was further limited in assessing the impact of momentary exposure to e-cigarette adverts. The results may therefore provide an underestimation of the true effects of e-cigarette advertising which is more dynamic and pervasive in everyday settings (e.g., billboards, posters, internet). Future research should examine other forms of e-cigarette advertising, and use a longitudinal design to corroborate the present findings. Further research is also warranted on the link between exposure to e-cigarette adverts, attitudes towards the tobacco industry and support for tobacco control policies.

Policy Implications

For peer review only

Our findings suggest that policies regarding e-cigarette advertising need to take into account the potential adverse cross-cueing effects on tobacco smoking among children. The present study coupled with two previous studies that have examined perceptions of the harms of tobacco smoking following exposure to e-cigarette adverts among children suggests the need to re-examine current regulations on advertising. E-cigarette advertising in the European Union (EU) is currently subsumed under the new Tobacco Products Directive (TPD).[40] These recent regulations limit the exposure of children to TV and newspaper e-cigarette advertising. However, the implementation of these regulations across EU member states still allows some form of e-cigarette advertising (posters, leaflets, billboards in shops), so children are still exposed to e-cigarette adverts. The TPD also does not explicitly prohibit the use of advertising themes/content that may be particularly appealing to children (such as flavoured, or glamorous e-cigarette adverts). Likewise, in the USA, the Food and Drug Administration recently began regulating e-cigarettes, but these regulations do not include provisions to curb children's exposure to e-cigarette advertising or to restrict e-cigarette adverts with potentially youth-appealing themes/content.[41]

CONCLUSIONS

This study adds to existing evidence that exposure to e-cigarette adverts reduces children's perceptions of the harm of occasional tobacco smoking. Further studies are warranted, using longitudinal and experimental designs, to assess a wider range of possible impacts of the marketing of e-cigarettes including attitudes towards the tobacco industry and tobacco control policies.

For peer review only

Acknowledgments: We would like to thank Emma Cartwright, Catherine Galloway, and Zorana Zupan for their assistance with data collection. We would also like to thank all participating children, and the teachers who assisted with the project.

Conflict of Interest: The authors have no conflicts of interest relevant to this article to disclose.

Funding Source and Financial Disclosure: The study was funded by the Department of Health Policy Research Programme (Policy Research Unit in Behaviour and Health [PR-UN-0409-10109]). The Department of Health had no role in the study design, data collection, analysis, or interpretation. The research was conducted independently of the funders, and the views expressed in this paper are those of the authors and not necessarily those of the Department of Health in England. The final version of the report and ultimate decision to submit for publication was determined by the authors.

Authors' contributions: All authors collaborated in designing the study. MV supervised the study and oversaw the acquisition of data. MV and DLC were responsible for the data analysis. All authors contributed in the interpretation of results. MV drafted the manuscript, ASJW, SC, DLC, SS, and TMM provided critical revisions to the manuscript. All authors read and approved the final version of the manuscript.

Data sharing statement: We are willing to make all data available to any interested parties. Please contact the corresponding author for more information.

For peer review only

REFERENCES

1. Petrescu DC, Vasiljevic M, Pepper JK, et al. What is the impact of e-cigarette adverts on children's perceptions of tobacco smoking? An experimental study. Tob Control 2017;**26**(4):421-27.

2. ASH. ASH Fact Sheet - Use of electronic cigarettes among children in Great Britain: Action on Smoking and Health, 2016:7.

3. Singh T, Arrazola R, Corey C, et al. Tobacco use among middle and high school students — United States, 2011–2015. MMWR Morb Mortal Wkly Rep: Centers for Disease Control and Prevention, 2016:361-7.

4. U.S. Department of Health and Human Services. E-cigarette use among youth and young adults. A report of the Surgeon General. Atlanta, GA: U.S: Department of Health and Human Services, Centers for Disease Control and Prevention, National Center for Chronic Disease Prevention and Health Promotion, Office on Smoking and Health, 2016.

5. McRobbie H, Bullen C, Hartmann-Boyce J, et al. Electronic cigarettes for smoking cessation and reduction. Cochrane Database Syst Rev 2014(12):CD010216.

6. Beard E, West R, Michie S, et al. Association between electronic cigarette use and changes in quit attempts, success of quit attempts, use of smoking cessation pharmacotherapy, and use of stop smoking services in England: time series analysis of population trends. BMJ 2016;**354**:i4645.

7. WHO. Electronic nicotine delivery systems: Report by WHO. Conference of the Parties to the WHO Framework Convention on Tobacco Control. Moscow, Russian Federation: World Health Organization Framework Convention on Tobacco Control, 2014:13.

8. Saebo G, Scheffels J. Assessing notions of denormalization and renormalization of smoking in light of e-cigarette regulation. Int J Drug Policy 2017;**49**(Supplement C):58-64.

9. Primack BA, Soneji S, Stoolmiller M, et al. Progression to traditional cigarette smoking after electronic cigarette use among US adolescents and young adults. JAMA Pediatr 2015;**169**(11):1018-23.

10. Leventhal AM, Strong DR, Kirkpatrick MG, et al. Association of electronic cigarette use with initiation of combustible tobacco product smoking in early adolescence. JAMA 2015;**314**(7):700-7.

11. Barrington-Trimis JL, Urman R, Berhane K, et al. E-Cigarettes and future cigarette use. Pediatrics 2016;**138**(1).

12. Best C, Haseen F, Currie D, et al. Relationship between trying an electronic cigarette and subsequent cigarette experimentation in Scottish adolescents: a cohort study. Tob Control 2017.

13. Conner M, Grogan S, Simms-Ellis R, et al. Do electronic cigarettes increase cigarette smoking in UK adolescents? Evidence from a 12-month prospective study. Tob Control 2017.

14. CRUK. Childhood smoking statistics: Cancer Research UK, 2016.

For peer review only

15. Farrelly MC, Duke JC, Crankshaw EC, et al. A randomized trial of the effect of e-cigarette TV advertisements on intentions to use e-cigarettes. Am J Prev Med 2015;**49**(5):686-93.

16. Vasiljevic M, Petrescu DC, Marteau TM. Impact of advertisements promoting candy-like flavoured e-cigarettes on appeal of tobacco smoking among children: an experimental study. Tob Control 2016;**25**(e2):e107-e12.

17. Sheeran P, Harris PR, Epton T. Does heightening risk appraisals change people's intentions and behavior? A meta-analysis of experimental studies. Psychol Bull 2014;**140**(2):511-43.

18. Brewer NT, Chapman GB, Gibbons FX, et al. Meta-analysis of the relationship between risk perception and health behavior: the example of vaccination. Health Psychol 2007;**26**(2):136-45.

19. Berg CJ, Romero DR, Pulvers K. Perceived harm of tobacco products and individual schemas of a smoker in relation to change in tobacco product use over one year among young adults. Subst Use Misuse 2015;**50**(1):90-8.

20. Choi WS, Harris KJ, Okuyemi K, et al. Predictors of smoking initiation among college-bound high school students. Ann Behav Med 2003;**26**(1):69-74.

21. Bjartveit K, Tverdal A. Health consequences of smoking 1-4 cigarettes per day. Tob Control 2005;**14**(5):315-20.

22. Song AV, Ling PM. Social smoking among young adults: investigation of intentions and attempts to quit. Am J Public Health 2011;**101**(7):1291-6.

23. Brown AE, Carpenter MJ, Sutfin EL. Occasional smoking in college: who, what, when and why? Addict Behav 2011;**36**(12):1199-204.

24. Arnett JJ. Optimistic bias in adolescent and adult smokers and nonsmokers. Addict Behav 2000;**25**(4):625-32.

25. Curry SJ, Grothaus L, McBride C. Reasons for quitting: intrinsic and extrinsic motivation for smoking cessation in a population-based sample of smokers. Addict Behav 1997;**22**(6):727-39.

26. Romer D, Jamieson P. Do adolescents appreciate the risks of smoking? Evidence from a national survey. J Adolesc Health 2001;**29**(1):12-21.

27. HSCIC. Smoking, drinking and drug use among young people in England—2014. Leeds: England: Health and Social Care Information Centre, 2015.

28. Wakefield M, Germain D, Durkin S, et al. An experimental study of effects on schoolchildren of exposure to point-of-sale cigarette advertising and pack displays. Health Educ Res 2006;**21**(3):338-47.

29. Emery S, Wakefield MA, Terry-McElrath Y, et al. Televised state-sponsored antitobacco advertising and youth smoking beliefs and behavior in the United States, 1999-2000. Arch Pediatr Adolesc Med 2005;**159**(7):639-45.

30. Pepper JK, Emery SL, Ribisl KM, et al. How risky is it to use e-cigarettes? Smokers' beliefs about their health risks from using novel and traditional tobacco products. J Behav Med 2015;**38**(2):318-26.

For peer review only

31. Chassin L, Presson CC, Sherman SJ, et al. Predicting the onset of cigarette smoking in adolescents: a longitudinal study1. J Appl Soc Psychol 1984;**14**(3):224-43.

32. Pierce JP, Choi WS, Gilpin EA, et al. Validation of susceptibility as a predictor of which adolescents take up smoking in the United States. Health Psychol 1996;**15**(5):355-61.

33. Unger JB, Johnson CA, Rohrbach LA. Recognition and liking of tobacco and alcohol advertisements among adolescents: relationships with susceptibility to substance use. Prev Med 1995;**24**(5):461-6.

34. Cumming G. The new statistics: why and how. Psychol Sci 2014;**25**(1):7-29.

35. Sheeran P, Klein WM, Rothman AJ. Health behavior change: Moving from observation to intervention. Annu Rev Psychol 2017;**68**(1):573-600.

36. De Witt Huberts JC, Evers C, De Ridder DT. "Because I am worth it": a theoretical framework and empirical review of a justification-based account of self-regulation failure. Pers Soc Psychol Rev 2014;**18**(2):119-38.

37. Gartner CE. E-cigarettes and youth smoking: be alert but not alarmed. Tob Control 2017.

38. Diepeveen S, Ling T, Suhrcke M, et al. Public acceptability of government intervention to change health-related behaviours: a systematic review and narrative synthesis. BMC Public Health 2013;**13**:756.

39. Jeffery RW, Forster JL, Schmid TL, et al. Community attitudes toward public policies to control alcohol, tobacco, and high-fat food consumption. Am J Prev Med 1990;**6**(1):12-9.

40. TPD. Directive 2014/40/EU of the European Parliament and of the Council of 3 April 2014 on the approximation of the laws, regulations and administrative provisions of the Member States concerning the manufacture, presentation and sale of tobacco and related products and repealing Directive 2001/37/EC. In: Union TEPatCotE, ed. 2014/40/EU, 2014.

41. FDA. Deeming tobacco products to be subject to the Federal Food, Drug, and Cosmetic Act, as amended by the Family Smoking Prevention and Tobacco Control Act; regulations on the sale and distribution of tobacco products and required warning statements for tobacco products. In: Services DoHaH, ed. 81 ed: Food and Drug Administration, 2016:134.

For peer review only

Table 1.

Demographic and smoking-related characteristics of (a) all randomised participants and (b) final sample

Table 1(a). *All randomised participants (n = 1449)*

	E-cig Adverts (*n* = 714)	Control Adverts (*n* = 735)	Total (*n* = 1449)
Age - M (SD)	13.71 (1.40)	13.73 (1.33)	13.72 (1.37)
Gender - Male % (n)	48.5 (346)	50.1 (368)	49.3 (714)
Ethnicity - White % (n)	74.6 (533)	72.9 (536)	73.8 (1069)
Regular cigarette use - Yes % (n)	12.3 (88)	12.1 (89)	12.2 (177)
Cigarette experimentation - Yes % (n)	16.1 (115)	15.1 (111)	15.6 (226)
E-cigarette awareness - Yes % (n)	92.9 (663)	93.9 (690)	93.4 (1353)
E-cigarette use - Yes % (n)	19.9 (142)	21.1 (155)	20.5 (297)
Cigarette use first in dual use - % (n)	8.7 (62)	7.9 (58)	8.3 (120)
E-cigarette use first in dual use - % (n)	8.3 (59)	7.6 (56)	7.9 (115)

Table 1(b). *Final sample of non-smokers and non-users of e-cigarettes (n = 1057)*

	E-cig Adverts (*n* = 521)	Control Adverts (*n* = 536)	Total (*n* = 1057)
Age - M (SD)	13.46 (1.40)	13.50 (1.34)	13.48 (1.37)
Gender - Male % (n)	45.1 (235)	48.7 (261)	46.9 (496)
Ethnicity - White % (n)	74.9 (390)	73.1 (392)	74.0 (782)

For peer review only

Table 2.

Non-parametric descriptive statistics of outcome measures across experimental groups

	E-cig Adverts (*n* = 521)	Control Adverts (*n* = 536)
Perceived harm of occasional tobacco smoking	**508.69**	**546.84**
Perceived harm of tobacco smoking in general	525.10	529.84
Perceived harm of regular tobacco smoking	531.91	524.18
Perceived disease risk (regular smoking)	529.87	522.22
Perceived disease risk (occasional smoking)	540.36	512.05
Tobacco smoking prevalence estimates	521.96	513.12
Perceived harm of occasional e-cigarette use	527.49	530.47
Perceived harm of e-cigarette use in general	516.81	539.84
Susceptibility to tobacco smoking	42.4	37.9
Perceived harm of regular e-cigarette use	530.06	527.97
Perceived disease risk (regular e-cig use)	520.34	527.56
Perceived disease risk (occasional e-cig use)	523.22	526.74
E-cigarette use prevalence estimates	523.19	513.90
Susceptibility to e-cig use	50.1	49.8
Appeal of adverts	**426.32**	**628.80**
Interest in buying and trying advertised product	**393.83**	**660.39**

Note: Boldface indicates statistically significant differences between columns at $p<.05$.Mean ranks are shown for all variables apart from susceptibility to tobacco smoking and e-cigarette use which are binary variables and are denoted by percentages.

For peer review only

Table 3.

Descriptive statistics [Mean (SD)] of outcome measures across experimental groups

	E-cig Adverts<br>(*n* = 521)	Control Adverts<br>(*n* = 536)
Perceived harm of occasional tobacco smoking	**2.85 (.97)**	**2.97 (1.04)**
Perceived harm of tobacco smoking in general	4.75 (.78)	4.81 (.61)
Perceived harm of regular tobacco smoking	4.66 (.55)	4.64 (.57)
Perceived disease risk (regular smoking)	4.22 (.66)	4.19 (.69)
Perceived disease risk (occasional smoking)	2.86 (.92)	2.76 (.91)
Tobacco smoking prevalence estimates	30.52 (21.35)	30.20 (21.88)
Perceived harm of occasional e-cigarette use	2.04 (.99)	2.07 (1.03)
Perceived harm of e-cigarette use in general	3.19 (1.02)	3.27 (1.04)
Perceived harm of regular e-cigarette use	3.33 (1.05)	3.32 (1.06)
Perceived disease risk (regular e-cig use)	2.78 (1.06)	2.80 (1.04)
Perceived disease risk (occasional e-cig use)	1.99 (.97)	1.97 (.91)
E-cigarette use prevalence estimates	26.86 (23.13)	26.78 (24.02)
Appeal of adverts	**1.78 (.50)**	**2.13 (.49)**
Interest in buying and trying advertised product	**1.37 (.45)**	**1.79 (.48)**

Note: Boldface indicates statistically significant differences between columns at p<.05.

For peer review only

Figure Captions

Figure 1. Forest plot of meta-analysis of impact of exposure to e-cigarette adverts on the perception that occasional smoking of one or two cigarettes is not very dangerous (continuous outcome)

For peer review only

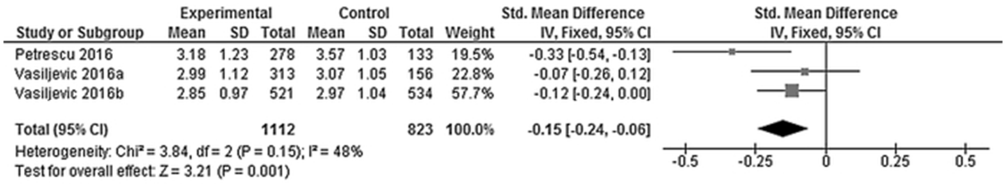

Figure 1. Forest plot of meta-analysis of impact of exposure to e-cigarette adverts on the perception that occasional smoking of one or two cigarettes is not very dangerous (continuous outcome)

29x5mm (600 x 600 DPI)

For peer review only

BMJ Open

E-cigarette adverts and children's perceptions of tobacco smoking harms: An experimental study and meta-analysis

Journal:	*BMJ Open*
Manuscript ID	bmjopen-2017-020247.R1
Article Type:	Research
Date Submitted by the Author:	27-Feb-2018

Complete List of Authors:	Vasiljevic, Milica; Behaviour and Health Research Unit<br>St John Wallis, Amelia; Behaviour and Health Research Unit<br>Codling, Saphsa; Behaviour and Health Research Unit<br>Couturier, Dominique-Laurent; University of Cambridge, Behaviour and Health Research Unit<br>Sutton, Stephen; University of Cambridge,<br>Marteau, Theresa; University of Cambridge, Behaviour and Health Research Unit

**Primary Subject Heading**:	Smoking and tobacco
Secondary Subject Heading:	Communication, Public health
Keywords:	PREVENTIVE MEDICINE, PUBLIC HEALTH, PRIORITY POPULATIONS, ELECTRONIC CIGARETTES, E-CIGARETTE MARKETING, TOBACCO SMOKING

SCHOLARONE™
Manuscripts

E-CIGARETTE ADVERTS AND CHILDREN'S PERCEPTIONS OF TOBACCO SMOKING HARMS: AN EXPERIMENTAL STUDY AND META-ANALYSIS

Milica Vasiljevic[a], PhD, Amelia St John Wallis[a], BA, Saphsa Codling[a], MA, Dominique-Laurent Couturier[a], PhD, Stephen Sutton[a,b], PhD, & Theresa M. Marteau[a], PhD

Affiliations: [a] Behaviour and Health Research Unit, Institute of Public Health, University of Cambridge, Cambridge, UK; and [b] Behavioural Science Group, Institute of Public Health, University of Cambridge, Cambridge, UK

Address correspondence to: Milica Vasiljevic, Behaviour and Health Research Unit, Institute of Public Health, Addenbrooke's Hospital, University of Cambridge, Cambridge, CB2 0SR, UK [milica.vasiljevic@medschl.cam.ac.uk], tel: +44 (0) 1223 761314.

Word Count: 4,054 (text only)

Number of Tables: 2

Number of Figures: 1

Abbreviations:

E-cig – e-cigarette

Advert – advertisement

For peer review only

ABSTRACT

Objectives: Children exposed to e-cigarette adverts may perceive occasional tobacco smoking as less harmful than children not exposed to e-cigarette adverts (Petrescu et al., 2016).[1] Given the potential cross-cueing effects of e-cigarette adverts on tobacco smoking there is an urgent need to establish whether the effect found in prior research is robust and replicable using a larger sample and a stronger control condition.

Design: A between-subjects experiment with one independent factor of two levels corresponding to the advertisements to which participants were exposed: glamorous adverts for e-cigarettes, or adverts for objects unrelated to smoking or vaping.

Participants: English school children aged 11-16 (*n* = 1449).

Outcomes: Perceived harm of occasional smoking of one or two tobacco cigarettes was the primary outcome. Secondary outcomes included: perceived harm of regular tobacco smoking, susceptibility to tobacco smoking and perceived prevalence of tobacco smoking in young people. Perceptions of using e-cigarettes were gauged by adapting all the outcome measures used to assess perceptions of tobacco smoking.

Results: Tobacco smokers and e-cigarette users were excluded from analyses (final sample *n* = 1057). Children exposed to glamorous e-cigarette adverts perceived the harms of occasional smoking of one or two tobacco cigarettes to be lower than those in the control group ($Z = -2.13$, $p = .033$). An updated meta-analysis comprising three studies with 1935 children confirmed that exposure to different types of e-cigarette adverts [glamorous, healthful, flavoured, non-flavoured] lowers the perceived harm of occasional smoking of one or two tobacco cigarettes ($Z = 3.21$, $p = .001$).

Conclusions: This study adds to existing evidence that exposure to e-cigarette adverts reduces children's perceptions of the harm of occasional tobacco smoking.

Keywords: advertising and promotion; electronic nicotine delivery devices; priority/special populations; non-cigarette tobacco products

For peer review only

STRENGTHS AND LIMITATIONS OF THIS STUDY:

- We replicate and extend prior findings regarding the perceived harm of occasional tobacco smoking using a larger sample and a stronger control condition.
- Meta-analysis of three studies confirms that exposing children to different e-cigarette adverts [glamorous, healthful, flavoured, or non-flavoured] lowers their perceived harm of occasional tobacco smoking.
- The present study was limited in several respects: the primary outcome measured perceived risk of smoking, not behaviour; and the design used only momentary exposure to e-cigarette adverts.

For peer review only

INTRODUCTION

Fewer children are smoking tobacco cigarettes today than several decades ago. However, the advent of electronic cigarettes (e-cigarettes) could disrupt this trend. The availability and use of e-cigarettes has risen rapidly in the last four years with an estimated 12%-24% of children aged 11-18 experimenting with e-cigarettes in the UK in 2015/16,[2] and 5.3% of middle schoolers and 16% of high schoolers in the USA in 2015.[3,4]

E-cigarettes have the potential for benefit and harm, the nature and scale of each being uncertain in the absence of much evidence. One potential benefit comes from providing a safe delivery mechanism for nicotine and an effective cessation aid. Evidence is accumulating to suggest that e-cigarettes can successfully be used as cessation aids by smokers.[5,6] Of concern, however, is their potential to make attitudes towards tobacco smoking more positive (i.e., to renormalise it) through, for example, marketing of objects that appear very similar to tobacco cigarettes that appeal to both adult and children non-smokers. Any such impact on children is of particular concern given the potential for any changes in attitudes to tobacco smoking to increase the chances of tobacco smoking in this group in particular. [4,7,8]

Several prospective studies in the USA and UK have found that among children e-cigarette use predicts tobacco smoking one year later.[9-13] By contrast, population level data show that the rising use and experimentation of e-cigarettes among children is accompanied by a continued decline in regular tobacco smoking in that group, from 15.8% to 9.2% amongst US high-schoolers in the period from 2011 to 2014,[3] and from 5% in 2010 to 3% in 2014 amongst 11-15 year olds in England.[14] Similar declines in rates of occasional (4% to 2%) and ever smoking

tobacco (25% to 18%) were recorded in England from 2010 to 2014.[14] Any impact on tobacco use of the recent upsurge in e-cigarette use in children will become more certain as the period of observation is extended. Experimental studies can also provide pertinent evidence.

The limited experimental evidence concerning the impact of e-cigarette exposure on children has focused on exposure to e-cigarette advertising. In one study, children exposed to televised e-cigarette adverts expressed more positive attitudes towards and greater intentions to use e-cigarettes.[15] In another study, children seeing candy flavoured e-cigarette adverts found these adverts more appealing and were more interested in buying and trying the products when compared to those children exposed to non-flavoured e-cigarette adverts.[16] But in neither study did exposure to e-cigarette advertisements significantly increase the appeal of smoking tobacco cigarettes. Only one study to date has found a cross-product influence of e-cigarette adverts on perceptions of the harms of occasional tobacco smoking.[1] In this study, exposing children to e-cigarette adverts characterised as depicting glamour or health had no significant impact on the appeal of smoking tobacco cigarettes, or the perceived harm of smoking more than 10 cigarettes per day. However, those exposed to either set of adverts perceived the harms of smoking one or two tobacco cigarettes occasionally to be lower than did those not exposed to any adverts.

Even though the size of the effect of perceived risk on routine or habitual behaviours is small to moderate,[17,18] it is nonetheless important in this context given the harms of tobacco smoking. Perceived harm (risk) of occasional smoking predicts tobacco smoking.[19,20] Furthermore, although the health consequences of occasional smoking can be as severe as regular smoking,[21] young smokers who smoke occasionally do not consider themselves smokers, believing they are immune to the risks associated with smoking, and have low intentions to quit.[22,23] In a similar

For peer review only

vein, perceived risk significantly predicts intentions and behaviours generally,[17,18] as well as more specifically in relation to smoking, with perceived harm associated with greater likelihood of staying abstinent or quitting if smoker.[24-26]

The aim of the present study is to replicate and extend recent findings showing that children perceive the harms of occasional tobacco smoking to be lower after exposure to e-cigarette adverts. By using a larger sample of children aged 11-16 and a control condition with equivalent task demands in which children were exposed to adverts for objects unrelated to tobacco smoking or vaping (pens), we sought to provide a more robust estimate of the effect found by Petrescu and colleagues.[1] In addition to assessing children's perceptions of the harms of occasional tobacco smoking, the present research also aimed to extend prior literature by examining children's perceptions of the harms of regular tobacco smoking, the perceived normativeness of tobacco smoking, and children's susceptibility to future tobacco smoking. In order to provide a more complete understanding of children's perceptions towards different nicotine products, we adapted all the measures assessing perceptions of tobacco smoking to also assess children's perceptions pertaining to e-cigarette use (including perceived harm, normativeness and potential susceptibility for future use).

For peer review only

METHODS

Design

A between-subjects experiment with one independent factor of two levels corresponding to the advertisements to which participants were exposed:

A. Adverts depicting e-cigarette use as glamorous

B. Adverts for objects [pens] unrelated to tobacco smoking or vaping (control condition)

Participants

Data were collected from 1449 English school children aged between 11-16 years (sampled from three schools, two based in Cambridgeshire and one based in Hampshire). Data were collected and analysed between January and September 2016. Randomisation was successful: there were no significant differences between the two experimental groups on any of the demographic, smoking or e-cigarette use characteristics measured. Ever-smokers and ever-users of e-cigarettes were excluded from the analyses leaving a final sample of 1057 participants. Characteristics of the full and final samples are shown in Table 1a and 1b respectively. This sample size provided more than 90% power at $\alpha = .05$ to detect a small-sized effect ($d = 0.27$) of glamorous e-cigarette adverts upon the perceived harm of occasional tobacco smoking (based on a recent study by Petrescu et al),[1] allowing for a reduction in sample size caused by excluding children with prior tobacco smoking or e-cigarette use.[27]

====================== PLACE TABLE 1 HERE ======================

Intervention

Each experimental condition displayed 10 adverts, with the control condition showing adverts of pens, and the e-cigarette condition showing adverts associating e-cigarette use with glamour. The e-cigarette adverts were taken from Petrescu et al.[1] The e-cigarette adverts for that study were sampled from the Stanford Adverts Repository (http://tobacco.stanford.edu/tobacco_main/index.php). A subset of 40 possible e-cigarette adverts were pilot tested with 16 year olds. Ten adverts were selected based on ratings for their depiction of glamour (for more details see Petrescu et al).[1] The adverts for the control condition were

For peer review only

selected from a larger sample of pen adverts. The pen adverts were sourced online. Pen adverts were chosen as the control stimuli due to their similar shape and look to tobacco and e-cigarettes. Three authors (MV, ASJW, SC) selected pen adverts to match the content of the e-cigarette adverts, including the presence of a person (with four adverts showing a woman using a pen, four adverts showing a man using a pen, and two adverts with no person in the advert).

Measures

Primary outcome:

Perceived harm of occasional tobacco smoking was assessed by an item adapted from Wakefield et al.[28] *"How dangerous do you think it is to smoke one or two cigarettes occasionally?"* rated on a five point scale, *1 = Not very dangerous* to *5 = Very dangerous*.

Secondary outcomes:

Perceived harm of tobacco smoking regularly and in general was measured using two items.[28] "*Smoking can harm your health"* rated from *1 = Strongly disagree* to *5 = Strongly agree*, and *"How dangerous do you think it is to smoke more than 10 cigarettes a day?"* rated from *1 = Not very dangerous* to *5 = Very dangerous*. These were analysed separately as in previous studies.[28,29]

Perceived risk of developing tobacco related diseases was measured by items adapted from Pepper et al.[30] *"How likely do you think it is that smoking tobacco cigarettes more than 10 times a day regularly [smoking tobacco cigarettes once or twice occasionally] would cause you to develop each of the following in the next 10 years? (If you're not sure, please give us your best*

For peer review only

guess) (a) lung cancer, (b) heart disease and (c) mouth or throat cancer" Ratings were provided on scales from *1= Not at all likely* to *5 = Extremely likely.* Two separate composite indices were made for perceived risk from regular (α = .76) and occasional (α = .90) tobacco smoking respectively.

Prevalence estimates of tobacco smoking were given on an open-ended question: "*How many young people your age out of 100 do you think smoke tobacco cigarettes?"*.[31]

Susceptibility to tobacco smoking was measured using three items: *"Do you think you will be smoking tobacco cigarettes when you are 18 years old?"*; *"Do you think you will smoke a tobacco cigarette at any time during the next year?"* and *"If one of your friends offered you a tobacco cigarette, would you smoke it?"*.[32] Participants were categorised as susceptible if they did not respond *"definitely not"* to all three items.

Appeal of adverts was assessed by asking: *"How much do you like this advert (not the product)?"*.[33] Responses ranged from *1 = Not at all,* to *4 = A lot*. Responses to the 10 adverts had high internal consistency (α = .80) and were averaged into a single index.

Interest in buying and trying products displayed in the adverts was assessed with the item: *"Does this advert make you want to buy and try this product?"* with scores ranging from *1 = Not at all,* to *4 = Yes, a lot*.[33] Responses had high internal consistency across the 10 adverts and were averaged into a single index (α = .85).

For peer review only

Perceptions of e-cigarette use: All of the outcomes described above, gauging perceptions of tobacco smoking, were adapted to also assess perceptions of using e-cigarettes (including: perceived harm of occasional and regular/general use of e-cigarettes; perceived risk of developing tobacco related diseases by using e-cigarettes regularly/occasionally; prevalence estimates of e-cigarette use; and susceptibility to use e-cigarettes). The composite indices for perceived risk from regular (α = .93) and occasional (α = .95) e-cigarette use had good inter-item reliabilities.

<u>*Other measures*</u>

Tobacco smoking was measured with two items: *"Have you ever smoked a tobacco cigarette?"* and *"Have you ever tried tobacco cigarette smoking, even one or two puffs?"*.[32] Items assessing tobacco cigarette smoking were adapted to assess *use of e-cigarettes*: *"Have you ever used an e-cigarette?"* and *"In the past 30 days, on how many days did you use an e-cigarette?"* For dual users we also asked: *"If you are both smoking tobacco cigarettes and using e-cigarettes, which product did you start using first?"* Gender, age, and ethnicity were also recorded.

Procedure

University of Cambridge's Psychology Research Ethics Committee approved the study [PRE.2015.106]. Prior passive parental consent was obtained, and the head-teachers of the schools acted *in-loco parentis* during data collection. The schools sent parents of eligible children letters to their home addresses and e-mail accounts with the Information Sheet and Opt-out Consent Forms for the present study. Children who were opted-out from participating in the study took part in alternative lesson arrangements organised by the schools. Before commencing the

For peer review only

study children also verbally assented to participation. Participating children were then reminded that they could withdraw from the study at any point.

The study materials were presented in paper-pencil format, with each participant receiving a booklet corresponding to one of the two experimental conditions depending on randomisation. Participants in the e-cigarette and control adverts conditions were each exposed to a series of 10 print-adverts in their booklets. To ensure that participants engaged with the adverts, after each advert they were asked to rate the appeal of the advert, and their interest in buying and trying the product (see Measures). Children in both experimental conditions were told the study was about their views on e-cigarettes and tobacco cigarettes. Children completed the experimental booklets at their own pace, and exposure to the adverts was not timed. The order in which the adverts appeared was fixed across participants. Potential confusion between e-cigarettes and tobacco cigarettes was managed by: (a) presenting all items pertaining to tobacco cigarettes and e-cigarettes in two separate sections; (b) adding a heading at the beginning of each section informing participants that the next section will deal with either tobacco or e-cigarettes; (c) including a picture of a tobacco cigarette and a picture of an e-cigarette at the beginning of each section; and (d) including a definition of e-cigarettes before the presentation of adverts and before assessing e-cigarette related items.

Participants were randomly assigned to one of the two groups, using a pre-established random sequence generated by the statistical package R. Prior to the testing session the different versions of the booklets were arranged in the pre-randomised order and these booklets were then distributed during testing. Both experimenters and participating children were blinded to allocated randomisation (even though children were exposed to adverts, they only saw one type

For peer review only

of advert and were not aware of what kind of adverts the other children were shown). Experimenters made sure that participants finishing earlier than others remained seated until everyone had finished. Once participants had completed their questionnaires, they were provided with a verbal and written debrief about the nature of the study.

Patient and Public Involvement

Four children who were the same age as eligible participants were asked to comment on the questionnaire materials prior to testing. The children gave suggestions on how the materials could be edited to make them easier to understand for participating children. The children who piloted the materials were not involved in study recruitment and conduct. Participating children will be informed of the study results with a short summary message distributed via their schools.

Statistical Analysis

All analyses were conducted using SPSS (version 23), R (version 3.3.1), and Review Manager (version 5.3). Responses on the primary and secondary outcomes were not normally distributed. Subsequent analyses were therefore conducted using non-parametric statistical tests (Mann-Whitney *U*, Chi-Squared and ordinal regression) to test equality of the location parameter between treatment groups. To provide a summary of the effects of e-cigarette advertising on perceived harm of occasional tobacco smoking, we meta-analysed the present data and the results of two published studies that also examined the impact of different types of e-cigarette adverts on perceptions of tobacco harm.[34] We searched published records for studies that could be synthesised, so the meta-analysis provides an accurate representation of all evidence currently available to us (for more details on the search strategy used and the included/excluded studies for the meta-analysis please see Online Supplementary Materials). All measures, experimental

For peer review only

conditions, and sample size calculations are reported in the manuscript. Exploratory analyses were also conducted on the subsample made up of ever-smokers and ever-users of e-cigarettes in order to explore whether e-cigarette adverts will have similar effects in that subsample (please see Online Supplementary Materials).

RESULTS

Primary outcome

Perceived harm of occasional tobacco smoking: Children exposed to glamorous e-cigarette adverts (Mean Rank = 508.69) perceived the danger as lower than did the control group (Mean Rank = 546.84), (Mann-Whitney $U = 129045.500$, $Z = -2.129$, $p = .033$). Using ordinal regression (controlling for clustering at the level of school) replicated these results ($t = -2.131$, $p = .033$).

Secondary outcomes

There were no statistically significant differences between the experimental groups in the perceived harm of regular smoking and smoking in general; perceived risk of developing tobacco-related diseases due to regular and occasional smoking; perceived susceptibility to smoking tobacco cigarettes; or the prevalence estimates for tobacco smoking. Similarly, there were no statistically significant differences between the experimental groups in: perceived harm of using e-cigarettes occasionally, regularly, or in general; perceived risk of developing tobacco-related diseases due to regular and occasional use of e-cigarettes; perceived susceptibility to using e-cigarettes; or prevalence estimates for using e-cigarettes. Please see Table 2 for more details on these analyses.

===================== PLACE TABLE 2 HERE =====================

For peer review only

Children exposed to glamorous e-cigarette adverts (Mean Rank = 426.32) liked the adverts less than did those in the control group (Mean Rank = 628.80), (Mann-Whitney U = 86133.500, Z = -10.797, p < .001). Furthermore, children exposed to glamorous e-cigarette adverts (Mean Rank = 393.83) were less interested in buying and trying the products shown in the adverts than were those in the control group (Mean Rank = 660.39), (Mann-Whitney U = 69202.500, Z = -14.298, p < .001).

Meta-analysis

The same measure of perceived harm of occasional tobacco smoking was used in two other, similar studies (see Online Supplementary Materials for more details on the search strategy used to identify eligible studies for synthesis). These assessed the impact of exposure to candy-like flavoured and non-flavoured e-cigarette adverts,[16] and the impact of glamorous and healthful e-cigarette adverts.[1] Using results from these two studies and the current study, we conducted a meta-analysis (using Review Manager version 5.3) of the continuous outcome, comparing those exposed to any type of advert for e-cigarettes with those in the control groups.

Exposing children to adverts for e-cigarettes decreases their perceived harm of occasional tobacco smoking: SMD = -0.15, 95% CI [-0.24, -0.06], I^2 = 48%, Z = 3.21, p = .001 (see Figure 1). Similar results were obtained when dichotomising responses to this outcome (as in Petrescu et al.).[1]

====================== PLACE FIGURE 1 HERE =====================

DISCUSSION

For peer review only

Children exposed to e-cigarette adverts depicting glamour perceived the harms of smoking one or two tobacco cigarettes occasionally to be lower than did those exposed to unrelated adverts. These results corroborate previous findings.[1] An updated meta-analysis comprising three studies (including the present study) with 1935 children confirmed that exposure to different types of e-cigarette adverts [glamorous, healthful, flavoured, or non-flavoured] lowers the perceived harm of occasional smoking of one or two tobacco cigarettes. The current study also replicates previous findings that exposure to glamorous and other types of adverts does not affect children's perceptions of the (high) harm of regularly smoking more than 10 tobacco cigarettes per day.[1,16] Our findings suggest that exposure to adverts for e-cigarettes may lead to differences in how children perceive the harms of tobacco smoking.

The absence of a significant impact of viewing e-cigarette adverts upon perceptions of the harms associated with regularly smoking more than 10 tobacco cigarettes a day is encouraging [see also[1,16]]. However, the impact on perceived harms of occasional smoking is concerning given that such perceptions can predict subsequent smoking.[19,20] Young occasional smokers in particular do not consider themselves smokers, believing they are immune to the risks associated with smoking, with low intentions to quit.[22,23] The effect of e-cigarette adverts on perceived harms of occasional tobacco smoking is therefore both theoretically and empirically important, given that perceived harm (risk) is a key construct affecting health behaviour change in multiple theories of behaviour change [see[35]]. Furthermore, the observed differential effects on the perceived harms of occasional *vs.* regular smoking may provide an indication that the former behaviour may be easier to mentally 'justify', thereby providing another potential route to self-regulation failure.[36]

Interestingly, children perceived that the harm of occasional tobacco smoking was lower when they were exposed to e-cigarette adverts, even though they rated the e-cigarette adverts as significantly less appealing and professed a lower interest in buying and trying the e-cigarettes when compared to the pens shown in the control condition. These findings may have important ramifications for future research and policy, since they suggest that the cross-product impact of e-cigarette adverts may largely work via an unconscious, implicit route that may not necessarily affect self-reported explicit appeal, but may change perceptions of harm (risk) which feed into children's behavioural decisions. These hypotheses merit further testing.

In more general terms, the population consequences of our findings are currently unknown. Two sets of outcomes need to be considered. First, the possible impact on tobacco smoking and second the possible impact on attitudes towards tobacco smoking. First, a small change in perceived harm of occasional smoking and no change in the already high perceived harm of smoking 10 or more cigarettes on a regular basis, may have no impact on the likelihood that children smoke tobacco cigarettes. This is supported by the evidence that perceived harms of occasional tobacco smoking have a small to moderate effect on actual smoking.[19 ,20] It is also consistent with the evidence that despite exposure to adverts and vaping there is no corresponding increase in the overall rates of children smoking tobacco. Indeed, the decline in rates observed over the last two decades has continued.[27 ,37] Nonetheless, any impact of e-cigarette adverts on tobacco smoking in children demands attention from policy makers.

Second, a lower perceived harm of occasional smoking may lead to more positive attitudes towards tobacco smoking and the tobacco industry, which in turn may result in more negative attitudes towards tobacco control policies. In high income countries, public attitudes towards

tobacco control policies, particularly those targeting children, are currently very positive.[38 ,39] Such attitudes are important in supporting policy makers in implementing effective tobacco control policies. Any lessening of these positive attitudes towards tobacco control would be a concern.

Strengths and Limitations with Future Directions

The large sample of children, and the use of a control condition in which children were exposed to a battery of adverts of objects unrelated to tobacco cigarettes or e-cigarettes strengthen the conclusions that can be drawn from the present study. By using a control condition in which children were exposed to pen adverts we were able to isolate the effects of e-cigarette adverts, and conclude that findings of lowered harm of occasional tobacco smoking can be attributed to e-cigarette adverts and not to viewing adverts more generally. Another strength of the current study is its contribution to an updated meta-analysis providing the most robust evidence to date that e-cigarette adverts of different kinds [glamorous, healthful, flavoured, or non-flavoured] may have a cross-product influence in lowering children's perceptions of the harms of occasional tobacco smoking.

The study was limited in several respects. The primary outcome was a belief and not a behaviour. Future studies should examine whether perceptions of harm following exposure to e-cigarettes translates into actual smoking behaviour.

The between-subjects design allowed us to control for any possible carry over effects of the different types of adverts. But this design also limits our ability to account for baseline differences in susceptibility to future tobacco smoking. Future research might usefully

For peer review only

incorporate within-subjects designs or assess baseline levels of susceptibility to tobacco smoking which could be controlled for in subsequent analyses.

The study was further limited in assessing the impact of momentary exposure to e-cigarette adverts. The results may therefore provide an underestimation of the true effects of e-cigarette advertising which is more dynamic and pervasive in everyday settings (e.g., billboards, posters, internet). Future research should examine other forms of e-cigarette advertising, and use a longitudinal design to corroborate the present findings. Further research is also warranted on the link between exposure to e-cigarette adverts, attitudes towards the tobacco industry and support for tobacco control policies.

Field experiments would provide a useful complement to the present study, since it is unclear whether the present findings obtained via a survey administered in school are generalisable to the real world. Furthermore, it is possible that the adverse effects of e-cigarette advertising found in this study may be short-lived. Whether short exposure to e-cigarette adverts has long-term effects on perceived harms of occasional tobacco smoking can only be ascertained by assessing outcomes in the longer as well as shorter-term.

Policy Implications

Our findings suggest that policies regarding e-cigarette advertising need to take into account the potential adverse cross-cueing effects on tobacco smoking among children. The present study coupled with two previous studies that have examined perceptions of the harms of tobacco smoking following exposure to e-cigarette adverts among children suggests the need to re-examine current regulations on advertising. E-cigarette advertising in the European Union (EU) is

For peer review only

currently subsumed under the new Tobacco Products Directive (TPD).[40] These recent regulations limit the exposure of children to TV and newspaper e-cigarette advertising. However, the implementation of these regulations across EU member states still allows some form of e-cigarette advertising (posters, leaflets, billboards in shops), so children are still exposed to e-cigarette adverts. The TPD also does not explicitly prohibit the use of advertising themes/content that may be particularly appealing to children (such as flavoured, or glamorous e-cigarette adverts). Likewise, in the USA, the Food and Drug Administration recently began regulating e-cigarettes, but these regulations do not include provisions to curb children's exposure to e-cigarette advertising or to restrict e-cigarette adverts with potentially youth-appealing themes/content.[41]

CONCLUSIONS

This study adds to existing evidence that exposure to e-cigarette adverts reduces children's perceptions of the harm of occasional tobacco smoking. Further studies are warranted, using longitudinal and experimental designs, to assess a wider range of possible impacts of the marketing of e-cigarettes including attitudes towards the tobacco industry and tobacco control policies.

For peer review only

Acknowledgments: We would like to thank Emma Cartwright, Catherine Galloway, and Zorana Zupan for their assistance with data collection. We would also like to thank all participating children and the children who piloted the materials, as well as the teachers who assisted with the project.

Conflict of Interest: The authors have no conflicts of interest relevant to this article to disclose.

Funding Source and Financial Disclosure: The study was funded by the Department of Health Policy Research Programme (Policy Research Unit in Behaviour and Health [PR-UN-0409-10109]). The Department of Health had no role in the study design, data collection, analysis, or interpretation. The research was conducted independently of the funders, and the views expressed in this paper are those of the authors and not necessarily those of the Department of Health in England. The final version of the report and ultimate decision to submit for publication was determined by the authors.

Authors' contributions: All authors collaborated in designing the study. MV supervised the study and oversaw the acquisition of data. MV and DLC were responsible for the data analysis. All authors contributed to the interpretation of results. MV drafted the manuscript, ASJW, SC, DLC, SS, and TMM provided critical revisions to the manuscript. All authors read and approved the final version of the manuscript.

Data sharing statement: We are willing to make all data available to any interested parties. Please contact the corresponding author for more information.

For peer review only

REFERENCES

1. Petrescu DC, Vasiljevic M, Pepper JK, et al. What is the impact of e-cigarette adverts on children's perceptions of tobacco smoking? An experimental study. Tob Control 2017;**26**(4):421-27.

2. ASH. ASH Fact Sheet - Use of electronic cigarettes among children in Great Britain: Action on Smoking and Health, 2016:7.

3. Singh T, Arrazola R, Corey C, et al. Tobacco use among middle and high school students — United States, 2011–2015. MMWR Morb Mortal Wkly Rep: Centers for Disease Control and Prevention, 2016:361-7.

4. U.S. Department of Health and Human Services. E-cigarette use among youth and young adults. A report of the Surgeon General. Atlanta, GA: U.S: Department of Health and Human Services, Centers for Disease Control and Prevention, National Center for Chronic Disease Prevention and Health Promotion, Office on Smoking and Health, 2016.

5. McRobbie H, Bullen C, Hartmann-Boyce J, et al. Electronic cigarettes for smoking cessation and reduction. Cochrane Database Syst Rev 2014(12):CD010216.

6. Beard E, West R, Michie S, et al. Association between electronic cigarette use and changes in quit attempts, success of quit attempts, use of smoking cessation pharmacotherapy, and use of stop smoking services in England: time series analysis of population trends. BMJ 2016;**354**:i4645.

7. WHO. Electronic nicotine delivery systems: Report by WHO. Conference of the Parties to the WHO Framework Convention on Tobacco Control. Moscow, Russian Federation: World Health Organization Framework Convention on Tobacco Control, 2014:13.

8. Saebo G, Scheffels J. Assessing notions of denormalization and renormalization of smoking in light of e-cigarette regulation. Int J Drug Policy 2017;**49**(Supplement C):58-64.

9. Primack BA, Soneji S, Stoolmiller M, et al. Progression to traditional cigarette smoking after electronic cigarette use among US adolescents and young adults. JAMA Pediatr 2015;**169**(11):1018-23.

10. Leventhal AM, Strong DR, Kirkpatrick MG, et al. Association of electronic cigarette use with initiation of combustible tobacco product smoking in early adolescence. JAMA 2015;**314**(7):700-7.

11. Barrington-Trimis JL, Urman R, Berhane K, et al. E-Cigarettes and future cigarette use. Pediatrics 2016;**138**(1).

12. Best C, Haseen F, Currie D, et al. Relationship between trying an electronic cigarette and subsequent cigarette experimentation in Scottish adolescents: a cohort study. Tob Control 2017.

13. Conner M, Grogan S, Simms-Ellis R, et al. Do electronic cigarettes increase cigarette smoking in UK adolescents? Evidence from a 12-month prospective study. Tob Control 2017.

14. CRUK. Childhood smoking statistics: Cancer Research UK, 2016.

For peer review only

15. Farrelly MC, Duke JC, Crankshaw EC, et al. A randomized trial of the effect of e-cigarette TV advertisements on intentions to use e-cigarettes. Am J Prev Med 2015;**49**(5):686-93.

16. Vasiljevic M, Petrescu DC, Marteau TM. Impact of advertisements promoting candy-like flavoured e-cigarettes on appeal of tobacco smoking among children: an experimental study. Tob Control 2016;**25**(e2):e107-e12.

17. Sheeran P, Harris PR, Epton T. Does heightening risk appraisals change people's intentions and behavior? A meta-analysis of experimental studies. Psychol Bull 2014;**140**(2):511-43.

18. Brewer NT, Chapman GB, Gibbons FX, et al. Meta-analysis of the relationship between risk perception and health behavior: the example of vaccination. Health Psychol 2007;**26**(2):136-45.

19. Berg CJ, Romero DR, Pulvers K. Perceived harm of tobacco products and individual schemas of a smoker in relation to change in tobacco product use over one year among young adults. Subst Use Misuse 2015;**50**(1):90-8.

20. Choi WS, Harris KJ, Okuyemi K, et al. Predictors of smoking initiation among college-bound high school students. Ann Behav Med 2003;**26**(1):69-74.

21. Bjartveit K, Tverdal A. Health consequences of smoking 1-4 cigarettes per day. Tob Control 2005;**14**(5):315-20.

22. Song AV, Ling PM. Social smoking among young adults: investigation of intentions and attempts to quit. Am J Public Health 2011;**101**(7):1291-6.

23. Brown AE, Carpenter MJ, Sutfin EL. Occasional smoking in college: who, what, when and why? Addict Behav 2011;**36**(12):1199-204.

24. Arnett JJ. Optimistic bias in adolescent and adult smokers and nonsmokers. Addict Behav 2000;**25**(4):625-32.

25. Curry SJ, Grothaus L, McBride C. Reasons for quitting: intrinsic and extrinsic motivation for smoking cessation in a population-based sample of smokers. Addict Behav 1997;**22**(6):727-39.

26. Romer D, Jamieson P. Do adolescents appreciate the risks of smoking? Evidence from a national survey. J Adolesc Health 2001;**29**(1):12-21.

27. HSCIC. Smoking, drinking and drug use among young people in England—2014. Leeds: England: Health and Social Care Information Centre, 2015.

28. Wakefield M, Germain D, Durkin S, et al. An experimental study of effects on schoolchildren of exposure to point-of-sale cigarette advertising and pack displays. Health Educ Res 2006;**21**(3):338-47.

29. Emery S, Wakefield MA, Terry-McElrath Y, et al. Televised state-sponsored antitobacco advertising and youth smoking beliefs and behavior in the United States, 1999-2000. Arch Pediatr Adolesc Med 2005;**159**(7):639-45.

30. Pepper JK, Emery SL, Ribisl KM, et al. How risky is it to use e-cigarettes? Smokers' beliefs about their health risks from using novel and traditional tobacco products. J Behav Med 2015;**38**(2):318-26.

For peer review only

31. Chassin L, Presson CC, Sherman SJ, et al. Predicting the onset of cigarette smoking in adolescents: a longitudinal study1. J Appl Soc Psychol 1984;**14**(3):224-43.

32. Pierce JP, Choi WS, Gilpin EA, et al. Validation of susceptibility as a predictor of which adolescents take up smoking in the United States. Health Psychol 1996;**15**(5):355-61.

33. Unger JB, Johnson CA, Rohrbach LA. Recognition and liking of tobacco and alcohol advertisements among adolescents: relationships with susceptibility to substance use. Prev Med 1995;**24**(5):461-6.

34. Cumming G. The new statistics: why and how. Psychol Sci 2014;**25**(1):7-29.

35. Sheeran P, Klein WM, Rothman AJ. Health behavior change: Moving from observation to intervention. Annu Rev Psychol 2017;**68**(1):573-600.

36. De Witt Huberts JC, Evers C, De Ridder DT. "Because I am worth it": a theoretical framework and empirical review of a justification-based account of self-regulation failure. Pers Soc Psychol Rev 2014;**18**(2):119-38.

37. Gartner CE. E-cigarettes and youth smoking: be alert but not alarmed. Tob Control 2017.

38. Diepeveen S, Ling T, Suhrcke M, et al. Public acceptability of government intervention to change health-related behaviours: a systematic review and narrative synthesis. BMC Public Health 2013;**13**:756.

39. Jeffery RW, Forster JL, Schmid TL, et al. Community attitudes toward public policies to control alcohol, tobacco, and high-fat food consumption. Am J Prev Med 1990;**6**(1):12-9.

40. TPD. Directive 2014/40/EU of the European Parliament and of the Council of 3 April 2014 on the approximation of the laws, regulations and administrative provisions of the Member States concerning the manufacture, presentation and sale of tobacco and related products and repealing Directive 2001/37/EC. In: Union TEPatCotE, ed. 2014/40/EU, 2014.

41. FDA. Deeming tobacco products to be subject to the Federal Food, Drug, and Cosmetic Act, as amended by the Family Smoking Prevention and Tobacco Control Act; regulations on the sale and distribution of tobacco products and required warning statements for tobacco products. In: Services DoHaH, ed. 81 ed: Food and Drug Administration, 2016:134.

For peer review only

Table 1(a).

Demographic and smoking-related characteristics of all randomised participants (n = 1449)

	**E-cig Adverts (*n* = 714)**	**Control Adverts (*n* = 735)**	**Test statistic**	***p*-value**	**Total (*n* = 1449)**
Age - *M (SD)*	13.71 (1.40)	13.73 (1.33)	.235	.815	13.72 (1.37)
Gender - Male % *(n)*	48.5 (346)	50.1 (368)	.933	.334	49.3 (714)
Ethnicity - White % *(n)*	74.6 (533)	72.9 (536)	.557	.456	73.8 (1069)
Regular cigarette use - Yes % *(n)*	12.3 (88)	12.1 (89)	.032	.858	12.2 (177)
Cigarette experimentation - Yes % *(n)*	16.1 (115)	15.1 (111)	.348	.555	15.6 (226)
E-cigarette awareness - Yes % *(n)*	92.9 (663)	93.9 (690)	.157	.692	93.4 (1353)
E-cigarette use - Yes % *(n)*	19.9 (142)	21.1 (155)	.230	.631	20.5 (297)
Cigarette use first in dual use - % *(n)*	8.7 (62)	7.9 (58)	.003	.956	8.3 (120)
E-cigarette use first in dual use - % *(n)*	8.3 (59)	7.6 (56)	.003	.956	7.9 (115)

Note: For all variables reported above differences between the experimental groups were examined using Chi-Squared tests, apart from the Age variable which was examined using an independent samples *t*-test.

For peer review only

Table 1(b).

Demographic characteristics of final sample of non-smokers and non-users of e-cigarettes (n = 1057)

	**E-cig Adverts (*n* = 521)**	**Control Adverts (*n* = 536)**	**Test statistic**	***p*-value**	**Total (*n* = 1057)**
Age - *M (SD)*	13.46 (1.40)	13.50 (1.34)	.472	.637	13.48 (1.37)
Gender - Male % *(n)*	45.1 (235)	48.7 (261)	3.147	.076	46.9 (496)
Ethnicity - White % *(n)*	74.9 (390)	73.1 (392)	.407	.524	74.0 (782)

Note: For all variables reported above differences between the experimental groups were examined using Chi-Squared tests, apart from the Age variable which was examined using an independent samples *t*-test.

For peer review only

Table 2.

Descriptive statistics of outcome measures across experimental groups

Outcome Variable	E-cig Adverts (*n* = 521)	Control Adverts (*n* = 536)	Test statistic	*p*-value
Perceived harm of occasional tobacco smoking	508.69	546.84	-2.129	.033
Perceived harm of tobacco smoking in general	525.10	529.84	-.435	.664
Perceived harm of regular tobacco smoking	531.91	524.18	-.512	.609
Perceived disease risk (regular smoking)	529.87	522.22	-.415	.678
Perceived disease risk (occasional smoking)	540.36	512.05	-1.524	.127
Tobacco smoking prevalence estimates	521.96	513.12	-.477	.634
Susceptibility to tobacco smoking	42.4	37.9	2.515	.113
Perceived harm of occasional e-cigarette use	527.49	530.47	-.167	.867
Perceived harm of e-cigarette use in general	516.81	539.84	-1.282	.200
Perceived harm of regular e-cigarette use	530.06	527.97	-.116	.908
Perceived disease risk (regular e-cig use)	520.34	527.56	-.389	.697
Perceived disease risk (occasional e-cig use)	523.22	526.74	-.193	.847
E-cigarette use prevalence estimates	523.19	513.90	-.501	.616
Susceptibility to e-cig use	50.1	49.8	.015	.902
Appeal of adverts	426.32	628.80	-10.797	<.001
Interest in buying and trying advertised product	393.83	660.39	-14.298	<.001

Note: For all outcome variables the test statistic corresponds to the *Z* value from the Mann Whitney *U* analyses (with corresponding Mean Ranks shown for each experimental group), except for the variables susceptibility to smoking and e-cigarettes use which are binary variables and are denoted by percentages summarised using the X^2 test statistic.

For peer review only

Figure Captions

Figure 1. Forest plot of meta-analysis of impact of exposure to e-cigarette adverts on the perception that occasional smoking of one or two cigarettes is not very dangerous (continuous outcome)

For peer review only

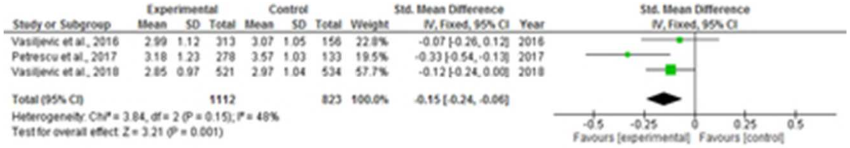

Figure 1. Forest plot of meta-analysis of impact of exposure to e-cigarette adverts on the perception that occasional smoking of one or two cigarettes is not very dangerous (continuous outcome)

35x6mm (300 x 300 DPI)

For peer review only

ONLINE SUPPLEMENTARY MATERIALS:

E-CIGARETTE ADVERTS AND CHILDREN'S PERCEPTIONS OF TOBACCO SMOKING HARMS: AN EXPERIMENTAL STUDY AND META-ANALYSIS

Milica Vasiljevic[a], PhD, Amelia St John Wallis[a], BA, Saphsa Codling[a], MA, Dominique-Laurent Couturier[a], PhD, Stephen Sutton[a,b], PhD, & Theresa M. Marteau[a], PhD

[a] Behaviour and Health Research Unit, Institute of Public Health, University of Cambridge, Cambridge, UK

[b] Behavioural Science Group, Institute of Public Health, University of Cambridge, Cambridge, UK

For peer review only

Appendix A: Exploratory Analyses..pp. 2-3

Appendix B: Details of searches for meta-analysis...........................pp. 4-8

For peer review only - http://bmjopen.bmj.com/site/about/guidelines.xhtml

Appendix A: Exploratory Analyses

Exploratory Analyses

We carried out exploratory analyses on the subsample of ever-smokers and ever-users of e-cigarettes (n = 362). We repeated all analyses carried out on the sample of non-smokers and non-e-cigarette users also in this subsample of ever-smokers and ever-users of e-cigarettes.

The only significant differences between the experimental conditions were on the indices of appeal (p = .037), and interest in buying and trying the products shown in the adverts (p = .032). No other effects reached the threshold of significance (see Table S1 below). These analyses should be considered with caution, since they are exploratory, and likely statistically underpowered given the sample size of ever smokers and e-cigarettes users is very small.

For peer review only - http://bmjopen.bmj.com/site/about/guidelines.xhtml

Table S1.

Descriptive statistics across experimental groups for ever-smokers and ever-users of e-cigarettes (Exploratory analyses)

**Outcome Variable**	**E-cig Adverts (*n* = 175)**	**Control Adverts (*n* = 187)**	**Test statistic**	***p*-value**
Perceived harm of occasional tobacco smoking	179.02	183.82	-.455	.649
Perceived harm of tobacco smoking in general	183.53	179.60	-.419	.675
Perceived harm of regular tobacco smoking	180.71	182.24	-.154	.878
Perceived disease risk (regular smoking)	182.85	180.24	-.240	.810
Perceived disease risk (occasional smoking)	177.62	183.23	-.516	.606
Tobacco smoking prevalence estimates	180.32	177.76	-.234	.815
Susceptibility to tobacco smoking	81.7	82.4	.376	.540
Perceived harm of occasional e-cigarette use	177.51	184.28	-.748	.454
Perceived harm of e-cigarette use in general	176.83	184.92	-.779	.436
Perceived harm of regular e-cigarette use	174.89	186.74	-1.125	.261
Perceived disease risk (regular e-cig use)	176.22	183.55	-.680	.496
Perceived disease risk (occasional e-cig use)	176.11	182.70	-.680	.497
E-cigarette use prevalence estimates	179.77	178.28	-.136	.892
Susceptibility to e-cig use	94.9	95.7	.022	.883
Appeal of adverts	169.65	192.59	-2.088	.037
Interest in buying and trying advertised product	169.33	192.89	-2.144	.032

Note: For all outcome variables the test statistic corresponds to the *Z* value from the Mann Whitney *U* analyses (with corresponding Mean Ranks shown for each experimental group), except for the variables susceptibility to smoking and e-cigarettes use which are binary variables and are denoted by percentages summarised using the X^2 test statistic.

For peer review only

Appendix B: Details of searches for meta-analysis

Eligibility criteria

Only randomised studies with any length of follow-up were included if they assessed exposure to e-cigarette adverts of any nature amongst children and adolescents. Eligible comparators were: (a) exposure to non-e-cigarette adverts; or (b) no exposure to adverts. Eligible studies also had to assess the effects of exposure in terms of the following outcome: perceived harm of occasional tobacco smoking. Studies that used non-randomised designs were not eligible. Studies that did not examine the effect of e-cigarette advertisements were also ineligible. Only studies reported in English were considered eligible. There were no eligibility restrictions for study publication status or date.

Search methods and study selection procedures

Eligible studies were located using electronic searches of PubMed and Google Scholar™. Keywords used in the database searches were combinations of the terms: 'e-cigarette adverts' (OR 'e-cigarette advertisements, OR 'e-cigarette marketing', OR 'electronic cigarette adverts', OR 'electronic cigarette advertisements, OR ' electronic cigarette marketing'), AND 'children' (OR 'adolescents'), AND 'perceived harm of occasional tobacco smoking' (OR 'harm of tobacco smoking', OR 'perceived harm of tobacco smoking'). Searches were conducted between 5 June and 17 July 2017 (and repeated between 25 January and 11 February 2018). Provisional eligibility decisions based on title-abstract screening were made by one reviewer (MV). Final eligibility decisions, based on examination of full-text study reports, were made by one reviewer (MV) and checked by a second (ASJW).

For peer review only - http://bmjopen.bmj.com/site/about/guidelines.xhtml

Data collection, risk of bias assessment and analysis

Data on the characteristics and results of included studies were extracted by one reviewer (MV) and checked by a second (ASJW). Study-level effect sizes were computed for the eligible outcome measure as the standardised mean difference (SMD) between comparison groups.

Study-level effect sizes were next combined using a fixed-effects meta-analysis due to the small sample size of identified eligible studies (k = 3), conducted using Review Manager 5.3. Statistical heterogeneity was assessed by inspection of graphical displays of each SMD and its 95% confidence interval, and a formal statistical test of homogeneity (I^2).

Results of the search

Bibliographic details of all studies identified by searches in PubMed and Google Scholar™ are provided below. Both sources yielded a total of eight primary study records (and one literature review). The eight primary study records were screened. Six primary studies were excluded based on screening, due to the studies not using randomised designs. Two studies were accepted for the meta-analysis (Petrescu et al., 2017; Vasiljevic et al., 2016), and were synthesised together with the primary data reported in the present manuscript (Vasiljevic et al., 2018). For characteristics of all three included studies in the meta-analysis see Table S2 below.

For peer review only - http://bmjopen.bmj.com/site/about/guidelines.xhtml

Table S2. Characteristics and results of included randomised controlled trials (*k* = 3).

Study	*Funding source*	*Design*	*Country, setting*	*Participants that completed study*	*Intervention(s)*	*Comparator(s)*	*Outcome measure (Perceived harm of occasional tobacco smoking)*	*Effect of exposure to e-cigarette adverts*	*Study-level effect size (SMD and 95% CI)*
Petrescu et al., 2017	Department of Health Policy Research Programme (Policy Research Unit in Behaviour and Health [PR-UN-0409-10109]).	RCT	UK, Home setting.	411 school children aged 11-16 years (M=13.09yrs, SD=1.68); 52.8% female.	Exposure to glamorous e-cigarette adverts; OR health-related e-cigarette adverts.	No e-cigarette adverts shown.	Single item rated on a five point scale, 1 = Not very dangerous to 5 = Very dangerous..	↓	-0.33 (-0.54 to -0.13)
Vasiljevic et al., 2016	Department of Health Policy Research Programme (Policy Research Unit in Behaviour and Health [PR-UN-0409-10109]).	RCT	UK, School setting.	471 school children aged 11-16 years (M=13.06yrs, SD=1.48); 48.2% female.	Exposure to candy flavoured e-cigarette adverts; OR non-candy flavoured e-cigarette adverts.	No e-cigarette adverts shown.	Single item rated on a five point scale, 1 = Not very dangerous to 5 = Very dangerous..	→←	-0.07 (-0.26 to 0.12)

For peer review only - http://bmjopen.bmj.com/site/about/guidelines.xhtml

Vasiljevic et al., 2018	Department of Health Policy Research Programme (Policy Research Unit in Behaviour and Health [PR-UN-0409-10109]).	RCT	UK, School setting.	1057 school children aged 11-16 years (M=13.48yrs, SD=1.37); 53.1% female.	Exposure to glamorous e-cigarette adverts. .	Exposure to pen adverts.	Single item rated on a five point scale, 1 = Not very dangerous to 5 = Very dangerous..	↓	-0.12 (-0.24 to 0.00)

For peer review only

Bibliographies of identified studies

Included studies

Petrescu et al., 2017

Petrescu DC, Vasiljevic M, Pepper JK, et al. What is the impact of e-cigarette adverts on children's perceptions of tobacco smoking? An experimental study. *Tob Control* 2017;26:421-27.

Vasiljevic et al., 2016

Vasiljevic M, Petrescu DC, Marteau TM. Impact of advertisements promoting candy-like flavoured e-cigarettes on appeal of tobacco smoking among children: an experimental study. *Tob Control* 2016;25(e2):e107-e12.

Vasiljevic et al., 2018

Vasiljevic M, St John Wallis A , Codling S, Couturier D-L, Sutton S, Marteau TM. E-cigarette adverts and children's perceptions of tobacco smoking harms: An experimental study. *BMJ Open* (under review).

Excluded study reports

Bauld L, Angus K, Ford A. Electronic Cigarette Marketing: Current Research and Policy. Cancer Research UK. 2016.

Conner M, Grogan S, Simms-Ellis R, Flett K, Sykes-Muskett B, Cowap L, Lawton R, Armitage CJ, Meads D, Torgerson C, West R. Do electronic cigarettes increase cigarette smoking in UK adolescents? Evidence from a 12-month prospective study. *Tob Control* 2017 doi:10.1136/tobaccocontrol-2016-053539.

Ford A, MacKintosh AM, Bauld L, Moodie C, Hastings G. Adolescents' responses to the promotion and flavouring of e-cigarettes. *Int J Public Health* 2016;61:215-24.

McKeganey N, Barnard M, Russell C. Visible vaping: E-cigarettes and the further de-normalization of smoking. *Int Arch Addict Res Med* 2016;2:1-6.

Pasch KE, Nicksic NE, Opara SC, Jackson C, Harrell MB, Perry CL. Recall of Point-of-Sale Marketing Predicts Cigar and E-Cigarette Use among Texas Youth. *Nicotine Tob Res* 2017 doi:10.1093/ntr/ntx237.

Popova L, So J, Sangalang A, Neilands TB, Ling PM. Do Emotions Spark Interest in Alternative Tobacco Products?. *Health Educ Behav* 2017;44:598-612.

Reinhold B, Fischbein R, Bhamidipalli SS, Bryant J, Kenne DR. Associations of attitudes towards electronic cigarettes with advertisement exposure and social determinants: a cross sectional study. *Tob Induc Dis* 2017;15:13.

For peer review only - http://bmjopen.bmj.com/site/about/guidelines.xhtml

CONSORT 2010 checklist of information to include when reporting a randomised trial*

Section/Topic	Item No	Checklist item	Reported on page No
**Title and abstract**			
	1a	Identification as a randomised trial in the title	1 (experiment)
	1b	Structured summary of trial design, methods, results, and conclusions (for specific guidance see CONSORT for abstracts)	2
**Introduction**			
Background and objectives	2a	Scientific background and explanation of rationale	4-6
	2b	Specific objectives or hypotheses	6-8
**Methods**			
Trial design	3a	Description of trial design (such as parallel, factorial) including allocation ratio	6-7
	3b	Important changes to methods after trial commencement (such as eligibility criteria), with reasons	n/a
Participants	4a	Eligibility criteria for participants	7
	4b	Settings and locations where the data were collected	10-12
Interventions	5	The interventions for each group with sufficient details to allow replication, including how and when they were actually administered	6-8; 10-12
Outcomes	6a	Completely defined pre-specified primary and secondary outcome measures, including how and when they were assessed	8-12
	6b	Any changes to trial outcomes after the trial commenced, with reasons	n/a
Sample size	7a	How sample size was determined	7
	7b	When applicable, explanation of any interim analyses and stopping guidelines	n/a
Randomisation:			
Sequence generation	8a	Method used to generate the random allocation sequence	11-12
	8b	Type of randomisation; details of any restriction (such as blocking and block size)	11-12
Allocation concealment mechanism	9	Mechanism used to implement the random allocation sequence (such as sequentially numbered containers), describing any steps taken to conceal the sequence until interventions were assigned	11-12
Implementation	10	Who generated the random allocation sequence, who enrolled participants, and who assigned participants to interventions	11-12
Blinding	11a	If done, who was blinded after assignment to interventions (for example, participants, care providers, those	10-12

For peer review only - http://bmjopen.bmj.com/site/about/guidelines.xhtml

		assessing outcomes) and how	
	11b	If relevant, description of the similarity of interventions	n/a
Statistical methods	12a	Statistical methods used to compare groups for primary and secondary outcomes	12
	12b	Methods for additional analyses, such as subgroup analyses and adjusted analyses	Online Supplements
**Results**			
Participant flow (a diagram is strongly recommended)	13a	For each group, the numbers of participants who were randomly assigned, received intended treatment, and were analysed for the primary outcome	7; 24-25
	13b	For each group, losses and exclusions after randomisation, together with reasons	7
Recruitment	14a	Dates defining the periods of recruitment and follow-up	7
	14b	Why the trial ended or was stopped	n/a
Baseline data	15	A table showing baseline demographic and clinical characteristics for each group	7; 24-25
Numbers analysed	16	For each group, number of participants (denominator) included in each analysis and whether the analysis was by original assigned groups	7; 12-14
Outcomes and estimation	17a	For each primary and secondary outcome, results for each group, and the estimated effect size and its precision (such as 95% confidence interval)	12-14; 26
	17b	For binary outcomes, presentation of both absolute and relative effect sizes is recommended	12-14; 26
Ancillary analyses	18	Results of any other analyses performed, including subgroup analyses and adjusted analyses, distinguishing pre-specified from exploratory	Online Supplements
Harms	19	All important harms or unintended effects in each group (for specific guidance see CONSORT for harms)	n/a
**Discussion**			
Limitations	20	Trial limitations, addressing sources of potential bias, imprecision, and, if relevant, multiplicity of analyses	3; 17-18
Generalisability	21	Generalisability (external validity, applicability) of the trial findings	14-19
Interpretation	22	Interpretation consistent with results, balancing benefits and harms, and considering other relevant evidence	14-16
**Other information**			
Registration	23	Registration number and name of trial registry	n/a
Protocol	24	Where the full trial protocol can be accessed, if available	n/a
Funding	25	Sources of funding and other support (such as supply of drugs), role of funders	20

*We strongly recommend reading this statement in conjunction with the CONSORT 2010 Explanation and Elaboration for important clarifications on all the items. If relevant, we also recommend reading CONSORT extensions for cluster randomised trials, non-inferiority and equivalence trials, non-pharmacological treatments, herbal interventions, and pragmatic trials. Additional extensions are forthcoming: for those and for up to date references relevant to this checklist, see www.consort-statement.org.

For peer review only - http://bmjopen.bmj.com/site/about/guidelines.xhtml

PRISMA 2009 Checklist

Section/topic	#	Checklist item	Reported on page #
**TITLE**			
Title	1	Identify the report as a systematic review, meta-analysis, or both.	1
**ABSTRACT**			
Structured summary	2	Provide a structured summary including, as applicable: background; objectives; data sources; study eligibility criteria, participants, and interventions; study appraisal and synthesis methods; results; limitations; conclusions and implications of key findings; systematic review registration number.	2
**INTRODUCTION**			
Rationale	3	Describe the rationale for the review in the context of what is already known.	12; 14
Objectives	4	Provide an explicit statement of questions being addressed with reference to participants, interventions, comparisons, outcomes, and study design (PICOS).	Online Supplements
**METHODS**			
Protocol and registration	5	Indicate if a review protocol exists, if and where it can be accessed (e.g., Web address), and, if available, provide registration information including registration number.	n/a
Eligibility criteria	6	Specify study characteristics (e.g., PICOS, length of follow-up) and report characteristics (e.g., years considered, language, publication status) used as criteria for eligibility, giving rationale.	Online Supplements
Information sources	7	Describe all information sources (e.g., databases with dates of coverage, contact with study authors to identify additional studies) in the search and date last searched.	Online Supplements
Search	8	Present full electronic search strategy for at least one database, including any limits used, such that it could be repeated.	Online Supplements
Study selection	9	State the process for selecting studies (i.e., screening, eligibility, included in systematic review, and, if applicable, included in the meta-analysis).	Online Supplements
Data collection process	10	Describe method of data extraction from reports (e.g., piloted forms, independently, in duplicate) and any processes for obtaining and confirming data from investigators.	Online Supplements
Data items	11	List and define all variables for which data were sought (e.g., PICOS, funding sources) and any assumptions and simplifications made.	Online Supplements
Risk of bias in individual studies	12	Describe methods used for assessing risk of bias of individual studies (including specification of whether this was done at the study or outcome level), and how this information is to be used in any data synthesis.	n/a
Summary measures	13	State the principal summary measures (e.g., risk ratio, difference in means).	Online

For peer review only - http://bmjopen.bmj.com/site/about/guidelines.xhtml

			Supplements
Synthesis of results	14	Describe the methods of handling data and combining results of studies, if done, including measures of consistency (e.g., I^2) for each meta-analysis.	Online Supplements
Section/topic	#	Checklist item	Reported on page #
Risk of bias across studies	15	Specify any assessment of risk of bias that may affect the cumulative evidence (e.g., publication bias, selective reporting within studies).	n/a
Additional analyses	16	Describe methods of additional analyses (e.g., sensitivity or subgroup analyses, meta-regression), if done, indicating which were pre-specified.	n/a
**RESULTS**			
Study selection	17	Give numbers of studies screened, assessed for eligibility, and included in the review, with reasons for exclusions at each stage, ideally with a flow diagram.	Online Supplements
Study characteristics	18	For each study, present characteristics for which data were extracted (e.g., study size, PICOS, follow-up period) and provide the citations.	Online Supplements
Risk of bias within studies	19	Present data on risk of bias of each study and, if available, any outcome level assessment (see item 12).	n/a
Results of individual studies	20	For all outcomes considered (benefits or harms), present, for each study: (a) simple summary data for each intervention group (b) effect estimates and confidence intervals, ideally with a forest plot.	Online Supplements
Synthesis of results	21	Present results of each meta-analysis done, including confidence intervals and measures of consistency.	14
Risk of bias across studies	22	Present results of any assessment of risk of bias across studies (see Item 15).	n/a
Additional analysis	23	Give results of additional analyses, if done (e.g., sensitivity or subgroup analyses, meta-regression [see Item 16]).	n/a
**DISCUSSION**			
Summary of evidence	24	Summarize the main findings including the strength of evidence for each main outcome; consider their relevance to key groups (e.g., healthcare providers, users, and policy makers).	14-19
Limitations	25	Discuss limitations at study and outcome level (e.g., risk of bias), and at review-level (e.g., incomplete retrieval of identified research, reporting bias).	Online Supplements
Conclusions	26	Provide a general interpretation of the results in the context of other evidence, and implications for future research.	14-19
**FUNDING**			
Funding	27	Describe sources of funding for the systematic review and other support (e.g., supply of data); role of funders for the systematic review.	20

For peer review only - http://bmjopen.bmj.com/site/about/guidelines.xhtml

BMJ Open

E-cigarette adverts and children's perceptions of tobacco smoking harms: An experimental study and meta-analysis

Journal:	*BMJ Open*
Manuscript ID	bmjopen-2017-020247.R2
Article Type:	Research
Date Submitted by the Author:	06-May-2018
Complete List of Authors:	Vasiljevic, Milica; Behaviour and Health Research Unit<br>St John Wallis, Amelia; Behaviour and Health Research Unit<br>Codling, Saphsa; Behaviour and Health Research Unit<br>Couturier, Dominique-Laurent; University of Cambridge, Behaviour and Health Research Unit<br>Sutton, Stephen; University of Cambridge,<br>Marteau, Theresa; University of Cambridge, Behaviour and Health Research Unit
**Primary Subject Heading**:	Smoking and tobacco
Secondary Subject Heading:	Communication, Public health
Keywords:	PREVENTIVE MEDICINE, PUBLIC HEALTH, PRIORITY POPULATIONS, ELECTRONIC CIGARETTES, E-CIGARETTE MARKETING, TOBACCO SMOKING

SCHOLARONE™
Manuscripts

E-CIGARETTE ADVERTS AND CHILDREN'S PERCEPTIONS OF TOBACCO SMOKING HARMS: AN EXPERIMENTAL STUDY AND META-ANALYSIS

Milica Vasiljevic[a], PhD, Amelia St John Wallis[a], BA, Saphsa Codling[a], MA, Dominique-Laurent Couturier[a], PhD, Stephen Sutton[a,b], PhD, & Theresa M. Marteau[a], PhD

Affiliations: [a] Behaviour and Health Research Unit, Institute of Public Health, University of Cambridge, Cambridge, UK; and [b] Behavioural Science Group, Institute of Public Health, University of Cambridge, Cambridge, UK

Address correspondence to: Milica Vasiljevic, Behaviour and Health Research Unit, Institute of Public Health, Addenbrooke's Hospital, University of Cambridge, Cambridge, CB2 0SR, UK [milica.vasiljevic@medschl.cam.ac.uk], tel: +44 (0) 1223 761314.

For peer review only

Word Count: 4,183 (text only)

Number of Tables: 2

Number of Figures: 1

Abbreviations:

E-cig – e-cigarette

Advert – advertisement

ABSTRACT

Objectives: Children exposed to e-cigarette adverts may perceive occasional tobacco smoking as less harmful than children not exposed to e-cigarette adverts. Given the potential cross-cueing effects of e-cigarette adverts on tobacco smoking there is an urgent need to establish whether the effect found in prior research is robust and replicable using a larger sample and a stronger control condition.

Design: A between-subjects experiment with one independent factor of two levels corresponding to the advertisements to which participants were exposed: glamorous adverts for e-cigarettes, or adverts for objects unrelated to smoking or vaping.

Participants: English school children aged 11-16 (n = 1449).

Outcomes: Perceived harm of occasional smoking of one or two tobacco cigarettes was the primary outcome. Secondary outcomes included: perceived harm of regular tobacco smoking, susceptibility to tobacco smoking and perceived prevalence of tobacco smoking in young people. Perceptions of using e-cigarettes were gauged by adapting all the outcome measures used to assess perceptions of tobacco smoking.

Results: Tobacco smokers and e-cigarette users were excluded from analyses (final sample n = 1057). Children exposed to glamorous e-cigarette adverts perceived the harms of occasional smoking of one or two tobacco cigarettes to be lower than those in the control group (Z = -2.13, p = .033). An updated meta-analysis comprising three studies with 1935 children confirmed that exposure to different types of e-cigarette adverts [glamorous, healthful, flavoured, non-flavoured] lowers the perceived harm of occasional smoking of one or two tobacco cigarettes (Z = 3.21, p = .001).

Conclusions: This study adds to existing evidence that exposure to e-cigarette adverts reduces children's perceptions of the harm of occasional tobacco smoking.

Keywords: advertising and promotion; electronic nicotine delivery devices; priority/special populations; non-cigarette tobacco products

For peer review only

STRENGTHS AND LIMITATIONS OF THIS STUDY:

- We replicate and extend prior findings regarding the perceived harm of occasional tobacco smoking using a larger sample and a stronger control condition.
- Meta-analysis of three studies confirms that exposing children to different e-cigarette adverts [glamorous, healthful, flavoured, or non-flavoured] lowers their perceived harm of occasional tobacco smoking.
- The present study was limited in several respects: the primary outcome measured perceived risk of smoking, not behaviour; and the design used only momentary exposure to e-cigarette adverts.

For peer review only

INTRODUCTION

Fewer children are smoking tobacco cigarettes today than several decades ago. However, the advent of electronic cigarettes (e-cigarettes) could disrupt this trend. The availability and use of e-cigarettes has risen rapidly in the last six years with an estimated 12%-24% of children aged 11-18 experimenting at least once with e-cigarettes in Great Britain in 2015/16,[1] and 13.5% of middle schoolers and 37.7% of high schoolers in the USA in 2016.[2,3]

E-cigarettes have the potential for benefit and harm, the nature and scale of each being uncertain in the absence of much evidence. One potential benefit comes from providing a safe delivery mechanism for nicotine and an effective cessation aid. Evidence is accumulating to suggest that e-cigarettes can successfully be used as cessation aids by smokers.[4,5] Of concern, however, is their potential to make attitudes towards tobacco smoking more positive (*i.e.,* to renormalise it) through, for example, marketing of objects that appear very similar to tobacco cigarettes that appeal to both adult and children non-smokers. Any such impact on children is of particular concern given the potential for any changes in attitudes to tobacco smoking to increase the chances of tobacco smoking in this group in particular.[3,6,7]

Several prospective studies in the USA and UK have found that among children e-cigarette use predicts tobacco smoking one year later.[8-12] By contrast, population level data show that the rising use and experimentation of e-cigarettes among children is accompanied by a continued decline in regular tobacco smoking in that group, from 15.8% to 8% amongst US high-schoolers and from 4.3% to 2.2% amongst US middle schoolers in the period from 2011 to 2016,[2] and from 5% in 2011 to 3% in 2016 amongst 11-15 year olds in England.[13] Similar declines in rates of ever

For peer review only

smoking tobacco (25% to 19%) were recorded in England from 2011 to 2016, with no change in the rates of occasional smoking (4% both in 2011 and 2016).[13] Any impact on tobacco use of the recent upsurge in e-cigarette use in children will become more certain as the period of observation is extended. Experimental studies can also provide pertinent evidence.

The limited experimental evidence concerning the impact of e-cigarette exposure on children has focused on exposure to e-cigarette advertising. In one study, children exposed to televised e-cigarette adverts expressed more positive attitudes towards and greater intentions to use e-cigarettes.[14] In another study, children seeing candy flavoured e-cigarette adverts found these adverts more appealing and were more interested in buying and trying the products when compared to those children exposed to non-flavoured e-cigarette adverts.[15] But in neither study did exposure to e-cigarette advertisements significantly increase the appeal of smoking tobacco cigarettes. Only one study to date has found a cross-product influence of e-cigarette adverts on perceptions of the harms of occasional tobacco smoking.[16] In this study, exposing children to e-cigarette adverts characterised as depicting glamour or health had no significant impact on the appeal of smoking tobacco cigarettes, or the perceived harm of smoking more than 10 cigarettes per day. However, those exposed to either set of adverts perceived the harms of smoking one or two tobacco cigarettes occasionally to be lower than did those not exposed to any adverts.

Even though the size of the effect of perceived risk on routine or habitual behaviours is small to moderate,[17,18] it is nonetheless important in this context given the harms of tobacco smoking. Perceived harm (risk) of occasional smoking predicts tobacco smoking.[19,20] Furthermore, although the health consequences of occasional smoking can be as severe as regular smoking,[21] young smokers who smoke occasionally do not consider themselves smokers, believing they are

For peer review only

immune to the risks associated with smoking, and have low intentions to quit.[22,23] In a similar vein, perceived risk significantly predicts intentions and behaviours generally,[17,18] as well as more specifically in relation to smoking, with perceived harm associated with greater likelihood of staying abstinent or quitting if smoker.[24-26]

The aim of the present study is to replicate and extend recent findings showing that children perceive the harms of occasional tobacco smoking to be lower after exposure to e-cigarette adverts. By using a larger sample of children aged 11-16 and a control condition with equivalent task demands in which children were exposed to adverts for objects unrelated to tobacco smoking or vaping (pens), we sought to provide a more robust estimate of the effect found by Petrescu and colleagues.[16] In addition to assessing children's perceptions of the harms of occasional tobacco smoking, the present research also aimed to extend prior literature by examining children's perceptions of the harms of regular tobacco smoking, the perceived normativeness of tobacco smoking, and children's susceptibility to future tobacco smoking. In order to provide a more complete understanding of children's perceptions towards different nicotine products, we adapted all the measures assessing perceptions of tobacco smoking to also assess children's perceptions pertaining to e-cigarette use (including perceived harm, normativeness and potential susceptibility for future use).

METHODS

Design

A between-subjects experiment with one independent factor of two levels corresponding to the advertisements to which participants were exposed:

For peer review only

A. Adverts depicting e-cigarette use as glamorous

B. Adverts for objects [pens] unrelated to tobacco smoking or vaping (control condition)

Participants

Data were collected from 1449 English school children aged between 11-16 years (sampled from three schools, two based in Cambridgeshire and one based in Hampshire). Data were collected and analysed between January and September 2016. Randomisation was successful: there were no significant differences between the two experimental groups on any of the demographic, smoking or e-cigarette use characteristics measured. Ever-smokers and ever-users of e-cigarettes were excluded from the analyses leaving a final sample of 1057 participants. Characteristics of the full and final samples are shown in Table 1a and 1b respectively. This sample size provided more than 90% power at $\alpha = .05$ to detect a small-sized effect ($d = 0.27$) of glamorous e-cigarette adverts upon the perceived harm of occasional tobacco smoking (based on a recent study by Petrescu et al.),[16] allowing for a reduction in sample size caused by excluding children with prior tobacco smoking or e-cigarette use.[27]

====================== **PLACE TABLE 1 HERE** ======================

Intervention

Each experimental condition displayed 10 adverts, with the control condition showing adverts of pens, and the e-cigarette condition showing adverts associating e-cigarette use with glamour. The e-cigarette adverts were taken from Petrescu et al.[16] The e-cigarette adverts for that study were sampled from the Stanford Adverts Repository (http://tobacco.stanford.edu/tobacco_main/index.php). A subset of 40 possible e-cigarette adverts were pilot tested with 16 year olds. Ten adverts were selected based on ratings for their depiction

For peer review only

of glamour (for more details see Petrescu et al).[16] The adverts for the control condition were selected from a larger sample of pen adverts. The pen adverts were sourced online. Pen adverts were chosen as the control stimuli due to their similar shape and look to tobacco and e-cigarettes. Three authors (MV, ASJW, SC) selected pen adverts to match the content of the e-cigarette adverts, including the presence of a person (with four adverts showing a woman using a pen, four adverts showing a man using a pen, and two adverts with no person in the advert).

For peer review only

Measures

<u>*Primary outcome:*</u>

Perceived harm of occasional tobacco smoking was assessed by an item adapted from Wakefield et al.[28] *"How dangerous do you think it is to smoke one or two cigarettes occasionally?"* rated on a five point scale, *1 = Not very dangerous* to *5 = Very dangerous*.

<u>*Secondary outcomes:*</u>

Perceived harm of tobacco smoking regularly and in general was measured using two items.[28] "*Smoking can harm your health"* rated from *1 = Strongly disagree* to *5 = Strongly agree*, and *"How dangerous do you think it is to smoke more than 10 cigarettes a day?"* rated from *1 = Not very dangerous* to *5 = Very dangerous*. These were analysed separately as in previous studies.[28,29]

Perceived risk of developing tobacco related diseases was measured by items adapted from Pepper et al.[30] *"How likely do you think it is that smoking tobacco cigarettes more than 10 times a day regularly [smoking tobacco cigarettes once or twice occasionally] would cause you to*

develop each of the following in the next 10 years? (If you're not sure, please give us your best guess) (a) lung cancer, (b) heart disease and (c) mouth or throat cancer" Ratings were provided on scales from *1= Not at all likely* to *5 = Extremely likely.* Two separate composite indices were made for perceived risk from regular (α = .76) and occasional (α = .90) tobacco smoking respectively.

Prevalence estimates of tobacco smoking were given on an open-ended question: "*How many young people your age out of 100 do you think smoke tobacco cigarettes?"*.[31]

Susceptibility to tobacco smoking was measured using three items: *"Do you think you will be smoking tobacco cigarettes when you are 18 years old?"*; *"Do you think you will smoke a tobacco cigarette at any time during the next year?"* and *"If one of your friends offered you a tobacco cigarette, would you smoke it?"*.[32] Participants were categorised as susceptible if they did not respond *"definitely not"* to all three items.

Appeal of adverts was assessed by asking: *"How much do you like this advert (not the product)?"*.[33] Responses ranged from *1 = Not at all,* to *4 = A lot*. Responses to the 10 adverts had high internal consistency (α = .80) and were averaged into a single index.

Interest in buying and trying products displayed in the adverts was assessed with the item: *"Does this advert make you want to buy and try this product?"* with scores ranging from *1 = Not at all,* to *4 = Yes, a lot.*[33] Responses had high internal consistency across the 10 adverts and were averaged into a single index (α = .85).

For peer review only

Perceptions of e-cigarette use: All of the outcomes described above, gauging perceptions of tobacco smoking, were adapted to also assess perceptions of using e-cigarettes (including: perceived harm of occasional and regular/general use of e-cigarettes; perceived risk of developing tobacco related diseases by using e-cigarettes regularly/occasionally; prevalence estimates of e-cigarette use; and susceptibility to use e-cigarettes). The composite indices for perceived risk from regular ($\alpha = .93$) and occasional ($\alpha = .95$) e-cigarette use had good inter-item reliabilities.

<u>Other measures</u>

Tobacco smoking was measured with two items: *"Have you ever smoked a tobacco cigarette?"* and *"Have you ever tried tobacco cigarette smoking, even one or two puffs?"*.[32] Items assessing tobacco cigarette smoking were adapted to assess *use of e-cigarettes*: *"Have you ever used an e-cigarette?"* and *"In the past 30 days, on how many days did you use an e-cigarette?"* For dual users we also asked: *"If you are both smoking tobacco cigarettes and using e-cigarettes, which product did you start using first?"* Gender, age, and ethnicity were also recorded.

Procedure

University of Cambridge's Psychology Research Ethics Committee approved the study [PRE.2015.106]. Prior passive parental consent was obtained, and the head-teachers of the schools acted *in-loco parentis* during data collection. The schools sent parents of eligible children letters to their home addresses and e-mail accounts with the Information Sheet and Opt-out Consent Forms for the present study. Children who were opted-out from participating in the study took part in alternative lesson arrangements organised by the schools. Before commencing the

For peer review only

study children also verbally assented to participation. Participating children were then reminded that they could withdraw from the study at any point.

The study materials were presented in paper-pencil format, with each participant receiving a booklet corresponding to one of the two experimental conditions depending on randomisation. Participants in the e-cigarette and control adverts conditions were each exposed to a series of 10 print-adverts in their booklets. To ensure that participants engaged with the adverts, after each advert they were asked to rate the appeal of the advert, and their interest in buying and trying the product (see Measures). Children in both experimental conditions were told the study was about their views on e-cigarettes and tobacco cigarettes. Children completed the experimental booklets at their own pace, and exposure to the adverts was not timed. The order in which the adverts appeared was fixed across participants. Potential confusion between e-cigarettes and tobacco cigarettes was managed by: (a) presenting all items pertaining to tobacco cigarettes and e-cigarettes in two separate sections; (b) adding a heading at the beginning of each section informing participants that the next section will deal with either tobacco or e-cigarettes; (c) including a picture of a tobacco cigarette and a picture of an e-cigarette at the beginning of each section; and (d) including a definition of e-cigarettes before the presentation of adverts and before assessing e-cigarette related items.

Participants were randomly assigned to one of the two groups, using a pre-established random sequence generated by the statistical package R. Prior to the testing session the different versions of the booklets were arranged in the pre-randomised order and these booklets were then distributed during testing. Both experimenters and participating children were blinded to allocated randomisation (even though children were exposed to adverts, they only saw one type

For peer review only

of advert and were not aware of what kind of adverts the other children were shown). Experimenters made sure that participants finishing earlier than others remained seated until everyone had finished. Once participants had completed their questionnaires, they were provided with a verbal and written debrief about the nature of the study.

Patient and Public Involvement

Four children who were the same age as eligible participants were asked to comment on the questionnaire materials prior to testing. The children gave suggestions on how the materials could be edited to make them easier to understand for participating children. The children who piloted the materials were not involved in study recruitment and conduct. Participating children will be informed of the study results with a short summary message distributed via their schools.

Statistical Analysis

All analyses were conducted using SPSS (version 23), R (version 3.3.1), and Review Manager (version 5.3). Responses on the primary and secondary outcomes were not normally distributed. Subsequent analyses were therefore conducted using non-parametric statistical tests (Mann-Whitney *U*, Chi-Squared and ordinal regression) to test equality of the location parameter between treatment groups. To provide a summary of the effects of e-cigarette advertising on perceived harm of occasional tobacco smoking, we meta-analysed the present data and the results of two published studies that also examined the impact of different types of e-cigarette adverts on perceptions of tobacco harm.[34] We searched published records for studies that could be synthesised, so the meta-analysis provides an accurate representation of all evidence currently available to us (for more details on the search strategy used and the included/excluded studies for the meta-analysis please see Online Supplementary Materials). All measures, experimental

For peer review only - http://bmjopen.bmj.com/site/about/guidelines.xhtml

conditions, and sample size calculations are reported in the manuscript. Exploratory analyses were also conducted on the subsample made up of ever-smokers and ever-users of e-cigarettes in order to explore whether e-cigarette adverts will have similar effects in that subsample (please see Online Supplementary Materials). Additional exploratory analyses examined whether age, gender or ethnicity moderated the effects of experimental condition on the primary outcome of interest (these analyses can be seen in the Online Supplementary Materials).

RESULTS

Primary outcome

Perceived harm of occasional tobacco smoking: Children exposed to glamorous e-cigarette adverts (Mean Rank = 508.69) perceived the danger as lower than did the control group (Mean Rank = 546.84), (Mann-Whitney U = 129045.500, Z = -2.129, p = .033). Using ordinal regression (controlling for clustering at the level of school) replicated these results (t = -2.131, p = .033).

Secondary outcomes

There were no statistically significant differences between the experimental groups in the perceived harm of regular smoking and smoking in general; perceived risk of developing tobacco-related diseases due to regular and occasional smoking; perceived susceptibility to smoking tobacco cigarettes; or the prevalence estimates for tobacco smoking. Similarly, there were no statistically significant differences between the experimental groups in: perceived harm of using e-cigarettes occasionally, regularly, or in general; perceived risk of developing tobacco-related diseases due to regular and occasional use of e-cigarettes; perceived susceptibility to using

For peer review only

e-cigarettes; or prevalence estimates for using e-cigarettes. Please see Table 2 for more details on these analyses.

====================== **PLACE TABLE 2 HERE** ======================

Children exposed to glamorous e-cigarette adverts (Mean Rank = 426.32) liked the adverts less than did those in the control group (Mean Rank = 628.80), (Mann-Whitney $U = 86133.500$, $Z = -10.797$, $p < .001$). Furthermore, children exposed to glamorous e-cigarette adverts (Mean Rank = 393.83) were less interested in buying and trying the products shown in the adverts than were those in the control group (Mean Rank = 660.39), (Mann-Whitney $U = 69202.500$, $Z = -14.298$, $p < .001$).

Meta-analysis

The same measure of perceived harm of occasional tobacco smoking was used in two other, similar studies (see Online Supplementary Materials for more details on the search strategy used to identify eligible studies for synthesis). These assessed the impact of exposure to candy-like flavoured and non-flavoured e-cigarette adverts,[15] and the impact of glamorous and healthful e-cigarette adverts.[16] Using results from these two studies and the current study, we conducted a meta-analysis (using Review Manager version 5.3) of the continuous outcome, comparing those exposed to any type of advert for e-cigarettes with those in the control groups.

Exposing children to adverts for e-cigarettes decreased their perceived harm of occasional tobacco smoking: SMD = -0.15, 95% CI [-0.24, -0.06], $I^2 = 48\%$, Z = 3.21, $p = .001$ (see Figure 1). Similar results were obtained when dichotomising responses to this outcome (as in Petrescu et al.).[16]

For peer review only - http://bmjopen.bmj.com/site/about/guidelines.xhtml

======================= **PLACE FIGURE 1 HERE** ======================

DISCUSSION

Children exposed to e-cigarette adverts depicting glamour perceived the harms of smoking one or two tobacco cigarettes occasionally to be lower than did those exposed to unrelated adverts. These results corroborate previous findings.[16] An updated meta-analysis comprising three studies (including the present study) with 1935 children confirmed that exposure to different types of e-cigarette adverts [glamorous, healthful, flavoured, or non-flavoured] lowers the perceived harm of occasional smoking of one or two tobacco cigarettes. The current study also replicates previous findings that exposure to glamorous and other types of adverts does not affect children's perceptions of the (high) harm of regularly smoking more than 10 tobacco cigarettes per day.[15,16] Our findings suggest that exposure to adverts for e-cigarettes may lead to differences in how children perceive the harms of tobacco smoking.

The absence of a significant impact of viewing e-cigarette adverts upon perceptions of the harms associated with regularly smoking more than 10 tobacco cigarettes a day is encouraging [see also[15,16]]. However, the impact on perceived harms of occasional smoking is concerning given that such perceptions can predict subsequent smoking.[19,20] Young occasional smokers in particular do not consider themselves smokers, believing they are immune to the risks associated with smoking, with low intentions to quit.[22,23] The effect of e-cigarette adverts on perceived harms of occasional tobacco smoking is therefore both theoretically and empirically important, given that perceived harm (risk) is a key construct affecting health behaviour change in multiple theories of behaviour change [see[35]]. Furthermore, the observed differential effects on the perceived harms of occasional *vs.* regular smoking may provide an indication that the former

For peer review only

behaviour may be easier to mentally 'justify', thereby providing another potential route to self-regulation failure.[36]

Interestingly, children perceived that the harm of occasional tobacco smoking was lower when they were exposed to e-cigarette adverts, even though they rated the e-cigarette adverts as significantly less appealing and professed a lower interest in buying and trying the e-cigarettes when compared to the pens shown in the control condition. These findings may have important ramifications for future research and policy, since they suggest that the cross-product impact of e-cigarette adverts may largely work via an unconscious, implicit route that may not necessarily affect self-reported explicit appeal, but may change perceptions of harm (risk) which feed into children's behavioural decisions. These hypotheses merit further testing.

In more general terms, the population consequences of our findings are currently unknown. Two sets of outcomes need to be considered. First, the possible impact on tobacco smoking and second the possible impact on attitudes towards tobacco smoking. First, a small change in perceived harm of occasional smoking and no change in the already high perceived harm of smoking 10 or more cigarettes on a regular basis, may have no impact on the likelihood that children smoke tobacco cigarettes. This is supported by the evidence that perceived harms of occasional tobacco smoking have a small to moderate effect on actual smoking.[19,20] It is also consistent with the evidence that despite exposure to adverts and vaping there is no corresponding increase in the overall rates of children smoking tobacco. Indeed, the decline in rates observed over the last two decades has continued.[13,27,37] Nonetheless, any impact of e-cigarette adverts on tobacco smoking in children demands attention from policy makers.

For peer review only

Second, a lower perceived harm of occasional smoking may lead to more positive attitudes towards tobacco smoking and the tobacco industry, which in turn may result in more negative attitudes towards tobacco control policies. In high income countries, public attitudes towards tobacco control policies, particularly those targeting children, are currently very positive.[38 ,39] Such attitudes are important in supporting policy makers in implementing effective tobacco control policies. Any lessening of these positive attitudes towards tobacco control would be a concern.

For peer review only

Strengths and Limitations with Future Directions

The large sample of children, and the use of a control condition in which children were exposed to a battery of adverts of objects unrelated to tobacco cigarettes or e-cigarettes strengthen the conclusions that can be drawn from the present study. By using a control condition in which children were exposed to pen adverts we were able to isolate the effects of e-cigarette adverts, and conclude that findings of lowered harm of occasional tobacco smoking can be attributed to e-cigarette adverts and not to viewing adverts more generally. Another strength of the current study is its contribution to an updated meta-analysis providing the most robust evidence to date that e-cigarette adverts of different kinds [glamorous, healthful, flavoured, or non-flavoured] may have a cross-product influence in lowering children's perceptions of the harms of occasional tobacco smoking.

The study was limited in several respects. The primary outcome was a belief and not a behaviour. Future studies should examine whether perceptions of harm following exposure to e-cigarettes translates into actual smoking behaviour.

The between-subjects design allowed us to control for any possible carry over effects of the different types of adverts. But this design also limits our ability to account for baseline differences in susceptibility to future tobacco smoking. Future research might usefully incorporate within-subjects designs or assess baseline levels of susceptibility to tobacco smoking which could be controlled for in subsequent analyses.

The study was further limited in assessing the impact of momentary exposure to e-cigarette adverts. The results may therefore provide an underestimation of the true effects of e-cigarette advertising which is more dynamic and pervasive in everyday settings (*e.g.,* billboards, posters, internet). Future research should examine other forms of e-cigarette advertising, and use a longitudinal design to corroborate the present findings. Further research is also warranted on the link between exposure to e-cigarette adverts, attitudes towards the tobacco industry and support for tobacco control policies.

Field experiments would provide a useful complement to the present study, since it is unclear whether the present findings obtained via a survey administered in schools are generalisable to the real world. Furthermore, it is possible that the adverse effects of e-cigarette advertising found in this study may be short-lived. Whether short exposure to e-cigarette adverts has long-term effects on perceived harms of occasional tobacco smoking can only be ascertained by assessing outcomes in the longer as well as shorter-term.

Policy Implications

Our findings suggest that policies regarding e-cigarette advertising need to take into account the potential adverse cross-cueing effects on tobacco smoking among children. The present study

For peer review only

coupled with two previous studies that have examined perceptions of the harms of tobacco smoking following exposure to e-cigarette adverts among children suggests the need to re-examine current regulations on advertising. E-cigarette advertising in the European Union (EU) is currently subsumed under the new Tobacco Products Directive (TPD).[40] These recent regulations limit the exposure of children to TV and newspaper e-cigarette advertising. However, the implementation of these regulations across EU member states still allows some form of e-cigarette advertising (posters, leaflets, billboards in shops), so children are still exposed to e-cigarette adverts. The TPD also does not explicitly prohibit the use of advertising themes/content that may be particularly appealing to children (such as flavoured, or glamorous e-cigarette adverts). Likewise, in the USA, the Food and Drug Administration recently began regulating e-cigarettes, but these regulations do not include provisions to curb children's exposure to e-cigarette advertising or to restrict e-cigarette adverts with potentially youth-appealing themes/content.[41]

CONCLUSIONS

This study adds to existing evidence that exposure to e-cigarette adverts reduces children's perceptions of the harm of occasional tobacco smoking. Further studies are warranted, using longitudinal and experimental designs, to assess a wider range of possible impacts of the marketing of e-cigarettes including attitudes towards the tobacco industry and tobacco control policies.

For peer review only

Acknowledgments: We would like to thank Emma Cartwright, Catherine Galloway, and Zorana Zupan for their assistance with data collection. We would also like to thank all participating children and the children who piloted the materials, as well as the teachers who assisted with the project.

Conflict of Interest: The authors have no conflicts of interest relevant to this article to disclose.

Funding Source and Financial Disclosure: This report is independent research commissioned and funded by the National Institute for Health Research Policy Research Programme (Policy Research Unit in Behaviour and Health [PR-UN-0409-10109]). The views expressed in this publication are those of the authors and not necessarily those of the NHS, the National Institute for Health Research, the Department of Health and Social Care or its arm's length bodies, and other Government Departments. The final version of the report and ultimate decision to submit for publication was determined by the authors.

Authors' contributions: All authors collaborated in designing the study. MV supervised the study and oversaw the acquisition of data. MV and DLC were responsible for the data analysis. All authors contributed to the interpretation of results. MV drafted the manuscript, ASJW, SC, DLC, SS, and TMM provided critical revisions to the manuscript. All authors read and approved the final version of the manuscript.

Data sharing statement: We are willing to make all data available to any interested parties. Please contact the corresponding author for more information.

REFERENCES

1. Action on Smoking and Health (ASH). *Use of electronic cigarettes among children in Great Britain*, 2016. https://casaa.org/wp-content/uploads/FS34Oct2016-ASH-UK-youth-use-stats.pdf

2. Jamal A, Gentzke A, Hu SS, et al. Tobacco Use Among Middle and High School Students — United States, 2011–2016. *MMWR Morb Mortal Wkly Rep* 2017;66:597–603. doi:10.15585/mmwr.mm6623a1

3. U.S. Department of Health and Human Services. *E-cigarette use among youth and young adults. A report of the Surgeon General.* Atlanta, GA: U.S: Department of Health and Human Services, Centers for Disease Control and Prevention, National Center for Chronic Disease Prevention and Health Promotion, Office on Smoking and Health, 2016. https://e-cigarettes.surgeongeneral.gov/documents/2016_sgr_full_report_non-508.pdf

4. McRobbie H, Bullen C, Hartmann-Boyce J, et al. Electronic cigarettes for smoking cessation and reduction. *Cochrane Database Syst Rev* 2014(12):CD010216. doi:10.1002/14651858.CD010216.pub2

5. Beard E, West R, Michie S, et al. Association between electronic cigarette use and changes in quit attempts, success of quit attempts, use of smoking cessation pharmacotherapy, and use of stop smoking services in England: time series analysis of population trends. *BMJ* 2016;354:i4645. doi:10.1136/bmj.i4645

6. WHO. *Electronic nicotine delivery systems: Report by WHO. Conference of the Parties to the WHO Framework Convention on Tobacco Control.* Moscow, Russian Federation: World Health Organization Framework Convention on Tobacco Control, 2014. http://apps.who.int/gb/fctc/PDF/cop6/FCTC_COP6_10Rev1-en.pdf

7. Saebo G, Scheffels J. Assessing notions of denormalization and renormalization of smoking in light of e-cigarette regulation. *Int J Drug Policy* 2017;49:58-64. doi:10.1016/j.drugpo.2017.07.026

8. Primack BA, Soneji S, Stoolmiller M, et al. Progression to traditional cigarette smoking after electronic cigarette use among US adolescents and young adults. *JAMA Pediatr* 2015;169:1018-23. doi:10.1001/jamapediatrics.2015.1742

9. Leventhal AM, Strong DR, Kirkpatrick MG, et al. Association of electronic cigarette use with initiation of combustible tobacco product smoking in early adolescence. *JAMA* 2015;314:700-7. doi:10.1001/jama.2015.8950

10. Barrington-Trimis JL, Urman R, Berhane K, et al. E-Cigarettes and future cigarette use. *Pediatrics* 2016;138:e20160379. doi:10.1542/peds.2016-0379

For peer review only

11. Best C, Haseen F, Currie D, et al. Relationship between trying an electronic cigarette and subsequent cigarette experimentation in Scottish adolescents: a cohort study. *Tob Control* 2017. doi:10.1136/tobaccocontrol-2017-053691

12. Conner M, Grogan S, Simms-Ellis R, et al. Do electronic cigarettes increase cigarette smoking in UK adolescents? Evidence from a 12-month prospective study. *Tob Control* 2017. doi:10.1136/tobaccocontrol-2016-053539

13. NHS Digital. *Smoking, drinking and drug use among young people in England—2016.* Richmond: England, 2017. https://files.digital.nhs.uk/publication/n/q/sdd-2016-rep.pdf

14. Farrelly MC, Duke JC, Crankshaw EC, et al. A randomized trial of the effect of e-cigarette TV advertisements on intentions to use e-cigarettes. *Am J Prev Med* 2015;49:686-93. doi:10.1016/j.amepre.2015.05.010

15. Vasiljevic M, Petrescu DC, Marteau TM. Impact of advertisements promoting candy-like flavoured e-cigarettes on appeal of tobacco smoking among children: an experimental study. *Tob Control* 2016;25:e107-e12. doi:10.1136/tobaccocontrol-2015-052593

16. Petrescu DC, Vasiljevic M, Pepper JK, et al. What is the impact of e-cigarette adverts on children's perceptions of tobacco smoking? An experimental study. *Tob Control* 2017;26:421-27. doi:10.1136/tobaccocontrol-2016-052940

17. Sheeran P, Harris PR, Epton T. Does heightening risk appraisals change people's intentions and behavior? A meta-analysis of experimental studies. *Psychol Bull* 2014;140:511-43. doi:10.1037/a0033065

18. Brewer NT, Chapman GB, Gibbons FX, et al. Meta-analysis of the relationship between risk perception and health behavior: the example of vaccination. *Health Psychol* 2007;26:136-45. doi:10.1037/0278-6133.26.2.136

19. Berg CJ, Romero DR, Pulvers K. Perceived harm of tobacco products and individual schemas of a smoker in relation to change in tobacco product use over one year among young adults. *Subst Use Misuse* 2015;50:90-8. doi:10.3109/10826084.2014.958858

20. Choi WS, Harris KJ, Okuyemi K, et al. Predictors of smoking initiation among college-bound high school students. *Ann Behav Med* 2003;26:69-74. doi:10.1207/S15324796ABM2601_09

21. Bjartveit K, Tverdal A. Health consequences of smoking 1-4 cigarettes per day. *Tob Control* 2005;14:315-20. doi:10.1136/tc.2005.011932

22. Song AV, Ling PM. Social smoking among young adults: investigation of intentions and attempts to quit. *Am J Public Health* 2011;101:1291-6. doi:10.2105/AJPH.2010.300012

For peer review only

23. Brown AE, Carpenter MJ, Sutfin EL. Occasional smoking in college: who, what, when and why? *Addict Behav* 2011;36:1199-204. doi:10.1016/j.addbeh.2011.07.024

24. Arnett JJ. Optimistic bias in adolescent and adult smokers and nonsmokers. *Addict Behav* 2000;25:625-32. doi:10.1016/S0306-4603(99)00072-6

25. Curry SJ, Grothaus L, McBride C. Reasons for quitting: intrinsic and extrinsic motivation for smoking cessation in a population-based sample of smokers. *Addict Behav* 1997;22:727-39. doi:10.1016/S0306-4603(97)00059-2

26. Romer D, Jamieson P. Do adolescents appreciate the risks of smoking? Evidence from a national survey. *J Adolesc Health* 2001;29:12-21. doi:10.1016/S1054-139X(01)00209-9

27. Health and Social Care Information Centre (HSCIC). *Smoking, drinking and drug use among young people in England—2014.* Leeds: England, 2015. http://www.hscic.gov.uk/pubs/sdd14

28. Wakefield M, Germain D, Durkin S, et al. An experimental study of effects on schoolchildren of exposure to point-of-sale cigarette advertising and pack displays. *Health Educ Res* 2006;21:338-47. doi:10.1093/her/cyl005

29. Emery S, Wakefield MA, Terry-McElrath Y, et al. Televised state-sponsored antitobacco advertising and youth smoking beliefs and behavior in the United States, 1999-2000. *Arch Pediatr Adolesc Med* 2005;159:639-45. doi:10.1001/archpedi.159.7.639

30. Pepper JK, Emery SL, Ribisl KM, et al. How risky is it to use e-cigarettes? Smokers' beliefs about their health risks from using novel and traditional tobacco products. *J Behav Med* 2015;38:318-26. doi:10.1007/s10865-014-9605-2

31. Chassin L, Presson CC, Sherman SJ, et al. Predicting the onset of cigarette smoking in adolescents: a longitudinal study. *J Appl Soc Psychol* 1984;14:224-43. doi: 10.1111/j.1559-1816.1984.tb02233.x

32. Pierce JP, Choi WS, Gilpin EA, et al. Validation of susceptibility as a predictor of which adolescents take up smoking in the United States. *Health Psychol* 1996;15:355-61. doi:10.1037/0278-6133.15.5.355

33. Unger JB, Johnson CA, Rohrbach LA. Recognition and liking of tobacco and alcohol advertisements among adolescents: relationships with susceptibility to substance use. *Prev Med* 1995;24:461-6. doi:10.1006/pmed.1995.1074

34. Cumming G. The new statistics: why and how. *Psychol Sci* 2014;25:7-29. doi:10.1177/0956797613504966

35. Sheeran P, Klein WM, Rothman AJ. Health behavior change: Moving from observation to intervention. *Annu Rev Psychol* 2017;68:573-600. doi:10.1146/annurev-psych-010416-044007

For peer review only - http://bmjopen.bmj.com/site/about/guidelines.xhtml

36. De Witt Huberts JC, Evers C, De Ridder DT. "Because I am worth it": a theoretical framework and empirical review of a justification-based account of self-regulation failure. *Pers Soc Psychol Rev* 2014;18:119-38. doi:10.1177/1088868313507533

37. Gartner CE. E-cigarettes and youth smoking: be alert but not alarmed. *Tob Control* 2017. doi:10.1136/tobaccocontrol-2017-054002

38. Diepeveen S, Ling T, Suhrcke M, et al. Public acceptability of government intervention to change health-related behaviours: a systematic review and narrative synthesis. *BMC Public Health* 2013;13:756. doi:10.1186/1471-2458-13-756

39. Jeffery RW, Forster JL, Schmid TL, et al. Community attitudes toward public policies to control alcohol, tobacco, and high-fat food consumption. *Am J Prev Med* 1990;6:12-9. doi:10.1016/S0749-3797(18)31039-0

40. European Parliament and Council. *Tobacco Products Directive - Directive 2014/40/EU of the European Parliament and of the Council.* http://ec.europa.eu/health/tobacco/docs/dir_201440_en.pdf

41. Food and Drug Administration (FDA). Deeming tobacco products to be subject to the Federal Food, Drug, and Cosmetic Act, as amended by the Family Smoking Prevention and Tobacco Control Act; regulations on the sale and distribution of tobacco products and required warning statements for tobacco products. *Fed Regist* 2016;81(28973):28973 -29106. https://federalregister.gov/a/2016-10685

For peer review only

Table 1(a).

Demographic and smoking-related characteristics of all randomised participants (n = 1449)

	**E-cig Adverts (*n* = 714)**	**Control Adverts (*n* = 735)**	**Test statistic**	***p*-value**	**Total (*n* = 1449)**
Age - *M (SD)*	13.71 (1.40)	13.73 (1.33)	.235	.815	13.72 (1.37)
Gender - Male % *(n)*	48.5 (346)	50.1 (368)	.933	.334	49.3 (714)
Ethnicity - White % *(n)*	74.6 (533)	72.9 (536)	.557	.456	73.8 (1069)
Regular cigarette use - Yes % *(n)*	12.3 (88)	12.1 (89)	.032	.858	12.2 (177)
Cigarette experimentation - Yes % *(n)*	16.1 (115)	15.1 (111)	.348	.555	15.6 (226)
E-cigarette awareness - Yes % *(n)*	92.9 (663)	93.9 (690)	.157	.692	93.4 (1353)
E-cigarette use - Yes % *(n)*	19.9 (142)	21.1 (155)	.230	.631	20.5 (297)
Cigarette use first in dual use - % *(n)*	8.7 (62)	7.9 (58)	.003	.956	8.3 (120)
E-cigarette use first in dual use - % *(n)*	8.3 (59)	7.6 (56)	.003	.956	7.9 (115)

Note: For all variables reported above differences between the experimental groups were examined using Chi-Squared tests, apart from the Age variable which was examined using an independent samples *t*-test.

For peer review only

Table 1(b).

Demographic characteristics of final sample of non-smokers and non-users of e-cigarettes (n = 1057)

	E-cig Adverts (*n* = 521)	Control Adverts (*n* = 536)	Test statistic	*p*-value	Total (*n* = 1057)
Age - *M (SD)*	13.46 (1.40)	13.50 (1.34)	.472	.637	13.48 (1.37)
Gender - Male % *(n)*	45.1 (235)	48.7 (261)	3.147	.076	46.9 (496)
Ethnicity - White % *(n)*	74.9 (390)	73.1 (392)	.407	.524	74.0 (782)

Note: For all variables reported above differences between the experimental groups were examined using Chi-Squared tests, apart from the Age variable which was examined using an independent samples *t*-test.

For peer review only

Table 2.

Descriptive statistics of outcome measures across experimental groups

**Outcome Variable**	**E-cig Adverts (*n* = 521)**	**Control Adverts (*n* = 536)**	**Test statistic**	***p*-value**
Perceived harm of occasional tobacco smoking	508.69	546.84	-2.129	.033
Perceived harm of tobacco smoking in general	525.10	529.84	-.435	.664
Perceived harm of regular tobacco smoking	531.91	524.18	-.512	.609
Perceived disease risk (regular smoking)	529.87	522.22	-.415	.678
Perceived disease risk (occasional smoking)	540.36	512.05	-1.524	.127
Tobacco smoking prevalence estimates	521.96	513.12	-.477	.634
Susceptibility to tobacco smoking	42.4	37.9	2.515	.113
Perceived harm of occasional e-cigarette use	527.49	530.47	-.167	.867
Perceived harm of e-cigarette use in general	516.81	539.84	-1.282	.200
Perceived harm of regular e-cigarette use	530.06	527.97	-.116	.908
Perceived disease risk (regular e-cig use)	520.34	527.56	-.389	.697
Perceived disease risk (occasional e-cig use)	523.22	526.74	-.193	.847
E-cigarette use prevalence estimates	523.19	513.90	-.501	.616
Susceptibility to e-cig use	50.1	49.8	.015	.902
Appeal of adverts	426.32	628.80	-10.797	<.001
Interest in buying and trying advertised product	393.83	660.39	-14.298	<.001

Note: For all outcome variables the test statistic corresponds to the *Z* value from the Mann Whitney *U* analyses (with corresponding Mean Ranks shown for each experimental group), except for the variables susceptibility to smoking and e-cigarettes use which are binary variables and are denoted by percentages summarised using the X^2 test statistic.

For peer review only

Figure Captions

Figure 1. Forest plot of meta-analysis of impact of exposure to e-cigarette adverts on the perception that occasional smoking of one or two cigarettes is not very dangerous (continuous outcome)

For peer review only

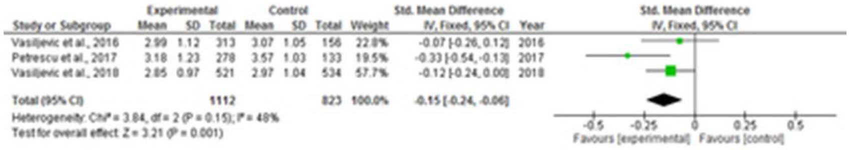

Figure 1. Forest plot of meta-analysis of impact of exposure to e-cigarette adverts on the perception that occasional smoking of one or two cigarettes is not very dangerous (continuous outcome)

35x6mm (300 x 300 DPI)

For peer review only

ONLINE SUPPLEMENTARY MATERIALS:

E-CIGARETTE ADVERTS AND CHILDREN'S PERCEPTIONS OF TOBACCO SMOKING HARMS: AN EXPERIMENTAL STUDY AND META-ANALYSIS

Milica Vasiljevic[a], PhD, Amelia St John Wallis[a], BA, Saphsa Codling[a], MA, Dominique-Laurent Couturier[a], PhD, Stephen Sutton[a,b], PhD, & Theresa M. Marteau[a], PhD

[a] Behaviour and Health Research Unit, Institute of Public Health, University of Cambridge, Cambridge, UK

[b] Behavioural Science Group, Institute of Public Health, University of Cambridge, Cambridge, UK

Appendix A: Exploratory Analyses .... pp. 2-6

Appendix B: Details of searches for meta-analysis .... pp. 7-11

For peer review only - http://bmjopen.bmj.com/site/about/guidelines.xhtml

Appendix A: Exploratory Analyses

Exploratory Analyses

We carried out two sets of exploratory analyses. First, multiple ordinal regressions (non-parametric) were conducted to examine any potential interactions between the experimental groups and age, gender, or ethnicity on the primary outcome (perceived harm of occasional tobacco smoking). The results of these ordinal regressions are reported in Tables S1 to S3 below. None of these three demographic variables moderated the effect of experimental group on perceived harm of occasional tobacco smoking (*ps* > .05).

Second, we carried out exploratory analyses on the subsample of ever-smokers and ever-users of e-cigarettes (n = 362). We repeated all analyses carried out on the sample of non-smokers and non-e-cigarette users also in this subsample of ever-smokers and ever-users of e-cigarettes.

The only significant differences between the experimental conditions were on the indices of appeal (p = .037), and interest in buying and trying the products shown in the adverts (p = .032). No other effects reached the threshold of significance (see Table S4 below). These analyses should be considered with caution, since they are exploratory, and likely statistically underpowered given the sample size of ever smokers and e-cigarettes users is very small.

For peer review only - http://bmjopen.bmj.com/site/about/guidelines.xhtml

Table S1.

Exploratory analyses of the interaction between experimental group and age on perceived harm of occasional tobacco smoking

**Variable**	**Estimate**	**S.E.**	***Z***	***p*-value**
Experimental Group	-0.21049	0.11536	-1.825	0.0681
Age	0.19659	0.08389	2.343	0.0191*
ExpGroup X Age	-0.19647	0.11555	-1.700	0.0891

Note. *denotes significance at <.05.

For peer review only

Table S2.

Exploratory analyses of the interaction between experimental group and gender on perceived harm of occasional tobacco smoking

**Variable**	**Estimate**	**S.E.**	***Z***	***p*-value**
Experimental Group	-0.13846	0.16358	-0.846	0.397
Gender	-0.09726	0.16356	-0.595	0.552
ExpGroup X Gender	-0.14149	0.23077	-0.613	0.540

For peer review only

For peer review only - http://bmjopen.bmj.com/site/about/guidelines.xhtml

Table S3.

Exploratory analyses of the interaction between experimental group and ethnicity on perceived harm of occasional tobacco smoking

Variable	Estimate	S.E.	*Z*	*p*-value
Experimental Group	-0.28055	0.13027	-2.154	0.0313*
Ethnicity	-0.09271	0.20358	-0.455	0.6488
ExpGroup X Ethnicity	0.24586	0.28323	0.868	0.3854

Note. *denotes significance at <.05.

For peer review only

For peer review only - http://bmjopen.bmj.com/site/about/guidelines.xhtml

Table S4.

Descriptive statistics across experimental groups for ever-smokers and ever-users of e-cigarettes (Exploratory analyses)

Outcome Variable	E-cig Adverts (*n* = 175)	Control Adverts (*n* = 187)	Test statistic	*p*-value
Perceived harm of occasional tobacco smoking	179.02	183.82	-.455	.649
Perceived harm of tobacco smoking in general	183.53	179.60	-.419	.675
Perceived harm of regular tobacco smoking	180.71	182.24	-.154	.878
Perceived disease risk (regular smoking)	182.85	180.24	-.240	.810
Perceived disease risk (occasional smoking)	177.62	183.23	-.516	.606
Tobacco smoking prevalence estimates	180.32	177.76	-.234	.815
Susceptibility to tobacco smoking	81.7	82.4	.376	.540
Perceived harm of occasional e-cigarette use	177.51	184.28	-.748	.454
Perceived harm of e-cigarette use in general	176.83	184.92	-.779	.436
Perceived harm of regular e-cigarette use	174.89	186.74	-1.125	.261
Perceived disease risk (regular e-cig use)	176.22	183.55	-.680	.496
Perceived disease risk (occasional e-cig use)	176.11	182.70	-.680	.497
E-cigarette use prevalence estimates	179.77	178.28	-.136	.892
Susceptibility to e-cig use	94.9	95.7	.022	.883
Appeal of adverts	169.65	192.59	-2.088	.037
Interest in buying and trying advertised product	169.33	192.89	-2.144	.032

Note: For all outcome variables the test statistic corresponds to the *Z* value from the Mann Whitney *U* analyses (with corresponding Mean Ranks shown for each experimental group), except for the variables susceptibility to smoking and e-cigarettes use which are binary variables and are denoted by percentages summarised using the X^2 test statistic.

For peer review only

Appendix B: Details of searches for meta-analysis

Eligibility criteria

Only randomised studies with any length of follow-up were included if they assessed exposure to e-cigarette adverts of any nature amongst children and adolescents. Eligible comparators were: (a) exposure to non-e-cigarette adverts; or (b) no exposure to adverts. Eligible studies also had to assess the effects of exposure in terms of the following outcome: perceived harm of occasional tobacco smoking. Studies that used non-randomised designs were not eligible. Studies that did not examine the effect of e-cigarette advertisements were also ineligible. Only studies reported in English were considered eligible. There were no eligibility restrictions for study publication status or date.

Search methods and study selection procedures

Eligible studies were located using electronic searches of PubMed and Google Scholar™. Keywords used in the database searches were combinations of the terms: 'e-cigarette adverts' (OR 'e-cigarette advertisements, OR 'e-cigarette marketing', OR 'electronic cigarette adverts', OR 'electronic cigarette advertisements, OR ' electronic cigarette marketing'), AND 'children' (OR 'adolescents'), AND 'perceived harm of occasional tobacco smoking' (OR 'harm of tobacco smoking', OR 'perceived harm of tobacco smoking'). Searches were conducted between 5 June and 17 July 2017 (and repeated between 25 January and 11 February 2018). Provisional eligibility decisions based on title-abstract screening were made by one reviewer (MV). Final eligibility decisions, based on examination of full-text study reports, were made by one reviewer (MV) and checked by a second (ASJW).

For peer review only - http://bmjopen.bmj.com/site/about/guidelines.xhtml

Data collection, risk of bias assessment and analysis

Data on the characteristics and results of included studies were extracted by one reviewer (MV) and checked by a second (ASJW). Study-level effect sizes were computed for the eligible outcome measure as the standardised mean difference (SMD) between comparison groups.

Study-level effect sizes were next combined using a fixed-effects meta-analysis due to the small sample size of identified eligible studies (k = 3), conducted using Review Manager 5.3. Statistical heterogeneity was assessed by inspection of graphical displays of each SMD and its 95% confidence interval, and a formal statistical test of homogeneity (I^2).

Results of the search

Bibliographic details of all studies identified by searches in PubMed and Google Scholar™ are provided below. Both sources yielded a total of eight primary study records (and one literature review). The eight primary study records were screened. Six primary studies were excluded based on screening, due to the studies not using randomised designs. Two studies were accepted for the meta-analysis (Petrescu et al., 2017; Vasiljevic et al., 2016), and were synthesised together with the primary data reported in the present manuscript (Vasiljevic et al., 2018). For characteristics of all three included studies in the meta-analysis see Table S5 below.

For peer review only - http://bmjopen.bmj.com/site/about/guidelines.xhtml

Table S5. Characteristics and results of included randomised controlled trials (*k* = 3).

Study	*Funding source*	*Design*	*Country, setting*	*Participants that completed study*	*Intervention(s)*	*Comparator(s)*	*Outcome measure (Perceived harm of occasional tobacco smoking)*	*Effect of exposure to e-cigarette adverts*	*Study-level effect size (SMD and 95% CI)*
Petrescu et al., 2017	Department of Health Policy Research Programme (Policy Research Unit in Behaviour and Health [PR-UN-0409-10109]).	RCT	UK, Home setting.	411 school children aged 11-16 years (M=13.09yrs, SD=1.68); 52.8% female.	Exposure to glamorous e-cigarette adverts; OR health-related e-cigarette adverts.	No e-cigarette adverts shown.	Single item rated on a five point scale, 1 = Not very dangerous to 5 = Very dangerous..	↓	-0.33 (-0.54 to -0.13)
Vasiljevic et al., 2016	Department of Health Policy Research Programme (Policy Research Unit in Behaviour and Health [PR-UN-0409-10109]).	RCT	UK, School setting.	471 school children aged 11-16 years (M=13.06yrs, SD=1.48); 48.2% female.	Exposure to candy flavoured e-cigarette adverts; OR non-candy flavoured e-cigarette adverts.	No e-cigarette adverts shown.	Single item rated on a five point scale, 1 = Not very dangerous to 5 = Very dangerous..	→←	-0.07 (-0.26 to 0.12)

For peer review only - http://bmjopen.bmj.com/site/about/guidelines.xhtml

Vasiljevic et al., 2018	Department of Health Policy Research Programme (Policy Research Unit in Behaviour and Health [PR-UN-0409-10109]).	RCT	UK, School setting.	1057 school children aged 11-16 years (M=13.48yrs, SD=1.37); 53.1% female.	Exposure to glamorous e-cigarette adverts. .	Exposure to pen adverts.	Single item rated on a five point scale, 1 = Not very dangerous to 5 = Very dangerous..	↓	-0.12 (-0.24 to 0.00)

For peer review only - http://bmjopen.bmj.com/site/about/guidelines.xhtml

Bibliographies of identified studies

Included studies

Petrescu et al., 2017

Petrescu DC, Vasiljevic M, Pepper JK, et al. What is the impact of e-cigarette adverts on children's perceptions of tobacco smoking? An experimental study. *Tob Control* 2017;26:421-27.

Vasiljevic et al., 2016

Vasiljevic M, Petrescu DC, Marteau TM. Impact of advertisements promoting candy-like flavoured e-cigarettes on appeal of tobacco smoking among children: an experimental study. *Tob Control* 2016;25(e2):e107-e12.

Vasiljevic et al., 2018

Vasiljevic M, St John Wallis A , Codling S, Couturier D-L, Sutton S, Marteau TM. E-cigarette adverts and children's perceptions of tobacco smoking harms: An experimental study. *BMJ Open* (under review).

Excluded study reports

Bauld L, Angus K, Ford A. *Electronic Cigarette Marketing: Current Research and Policy*. Cancer Research UK. 2016.

Conner M, Grogan S, Simms-Ellis R, Flett K, Sykes-Muskett B, Cowap L, Lawton R, Armitage CJ, Meads D, Torgerson C, West R. Do electronic cigarettes increase cigarette smoking in UK adolescents? Evidence from a 12-month prospective study. *Tob Control* 2017 doi:10.1136/tobaccocontrol-2016-053539.

Ford A, MacKintosh AM, Bauld L, Moodie C, Hastings G. Adolescents' responses to the promotion and flavouring of e-cigarettes. *Int J Public Health* 2016;61:215-24.

McKeganey N, Barnard M, Russell C. Visible vaping: E-cigarettes and the further de-normalization of smoking. *Int Arch Addict Res Med* 2016;2:1-6.

Pasch KE, Nicksic NE, Opara SC, Jackson C, Harrell MB, Perry CL. Recall of Point-of-Sale Marketing Predicts Cigar and E-Cigarette Use among Texas Youth. *Nicotine Tob Res* 2017 doi:10.1093/ntr/ntx237.

Popova L, So J, Sangalang A, Neilands TB, Ling PM. Do Emotions Spark Interest in Alternative Tobacco Products?. *Health Educ Behav* 2017;44:598-612.

Reinhold B, Fischbein R, Bhamidipalli SS, Bryant J, Kenne DR. Associations of attitudes towards electronic cigarettes with advertisement exposure and social determinants: a cross sectional study. *Tob Induc Dis* 2017;15:13.

For peer review only - http://bmjopen.bmj.com/site/about/guidelines.xhtml

CONSORT 2010 checklist of information to include when reporting a randomised trial*

Section/Topic	Item No	Checklist item	Reported on page No
**Title and abstract**			
	1a	Identification as a randomised trial in the title	1 (experiment)
	1b	Structured summary of trial design, methods, results, and conclusions (for specific guidance see CONSORT for abstracts)	2
**Introduction**			
Background and objectives	2a	Scientific background and explanation of rationale	4-6
	2b	Specific objectives or hypotheses	6-8
**Methods**			
Trial design	3a	Description of trial design (such as parallel, factorial) including allocation ratio	6-7
	3b	Important changes to methods after trial commencement (such as eligibility criteria), with reasons	n/a
Participants	4a	Eligibility criteria for participants	7
	4b	Settings and locations where the data were collected	10-12
Interventions	5	The interventions for each group with sufficient details to allow replication, including how and when they were actually administered	6-8; 10-12
Outcomes	6a	Completely defined pre-specified primary and secondary outcome measures, including how and when they were assessed	8-12
	6b	Any changes to trial outcomes after the trial commenced, with reasons	n/a
Sample size	7a	How sample size was determined	7
	7b	When applicable, explanation of any interim analyses and stopping guidelines	n/a
Randomisation:			
Sequence generation	8a	Method used to generate the random allocation sequence	11-12
	8b	Type of randomisation; details of any restriction (such as blocking and block size)	11-12
Allocation concealment mechanism	9	Mechanism used to implement the random allocation sequence (such as sequentially numbered containers), describing any steps taken to conceal the sequence until interventions were assigned	11-12
Implementation	10	Who generated the random allocation sequence, who enrolled participants, and who assigned participants to interventions	11-12
Blinding	11a	If done, who was blinded after assignment to interventions (for example, participants, care providers, those	10-12

For peer review only - http://bmjopen.bmj.com/site/about/guidelines.xhtml

		assessing outcomes) and how	
	11b	If relevant, description of the similarity of interventions	n/a
Statistical methods	12a	Statistical methods used to compare groups for primary and secondary outcomes	12
	12b	Methods for additional analyses, such as subgroup analyses and adjusted analyses	Online Supplements
**Results**			
Participant flow (a diagram is strongly recommended)	13a	For each group, the numbers of participants who were randomly assigned, received intended treatment, and were analysed for the primary outcome	7; 24-25
	13b	For each group, losses and exclusions after randomisation, together with reasons	7
Recruitment	14a	Dates defining the periods of recruitment and follow-up	7
	14b	Why the trial ended or was stopped	n/a
Baseline data	15	A table showing baseline demographic and clinical characteristics for each group	7; 24-25
Numbers analysed	16	For each group, number of participants (denominator) included in each analysis and whether the analysis was by original assigned groups	7; 12-14
Outcomes and estimation	17a	For each primary and secondary outcome, results for each group, and the estimated effect size and its precision (such as 95% confidence interval)	12-14; 26
	17b	For binary outcomes, presentation of both absolute and relative effect sizes is recommended	12-14; 26
Ancillary analyses	18	Results of any other analyses performed, including subgroup analyses and adjusted analyses, distinguishing pre-specified from exploratory	Online Supplements
Harms	19	All important harms or unintended effects in each group (for specific guidance see CONSORT for harms)	n/a
**Discussion**			
Limitations	20	Trial limitations, addressing sources of potential bias, imprecision, and, if relevant, multiplicity of analyses	3; 17-18
Generalisability	21	Generalisability (external validity, applicability) of the trial findings	14-19
Interpretation	22	Interpretation consistent with results, balancing benefits and harms, and considering other relevant evidence	14-16
**Other information**			
Registration	23	Registration number and name of trial registry	n/a
Protocol	24	Where the full trial protocol can be accessed, if available	n/a
Funding	25	Sources of funding and other support (such as supply of drugs), role of funders	20

*We strongly recommend reading this statement in conjunction with the CONSORT 2010 Explanation and Elaboration for important clarifications on all the items. If relevant, we also recommend reading CONSORT extensions for cluster randomised trials, non-inferiority and equivalence trials, non-pharmacological treatments, herbal interventions, and pragmatic trials. Additional extensions are forthcoming: for those and for up to date references relevant to this checklist, see www.consort-statement.org.

For peer review only

For peer review only - http://bmjopen.bmj.com/site/about/guidelines.xhtml

PRISMA 2009 Checklist

Section/topic	#	Checklist item	Reported on page #
**TITLE**			
Title	1	Identify the report as a systematic review, meta-analysis, or both.	1
**ABSTRACT**			
Structured summary	2	Provide a structured summary including, as applicable: background; objectives; data sources; study eligibility criteria, participants, and interventions; study appraisal and synthesis methods; results; limitations; conclusions and implications of key findings; systematic review registration number.	2
**INTRODUCTION**			
Rationale	3	Describe the rationale for the review in the context of what is already known.	12; 14
Objectives	4	Provide an explicit statement of questions being addressed with reference to participants, interventions, comparisons, outcomes, and study design (PICOS).	Online Supplements
**METHODS**			
Protocol and registration	5	Indicate if a review protocol exists, if and where it can be accessed (e.g., Web address), and, if available, provide registration information including registration number.	n/a
Eligibility criteria	6	Specify study characteristics (e.g., PICOS, length of follow-up) and report characteristics (e.g., years considered, language, publication status) used as criteria for eligibility, giving rationale.	Online Supplements
Information sources	7	Describe all information sources (e.g., databases with dates of coverage, contact with study authors to identify additional studies) in the search and date last searched.	Online Supplements
Search	8	Present full electronic search strategy for at least one database, including any limits used, such that it could be repeated.	Online Supplements
Study selection	9	State the process for selecting studies (i.e., screening, eligibility, included in systematic review, and, if applicable, included in the meta-analysis).	Online Supplements
Data collection process	10	Describe method of data extraction from reports (e.g., piloted forms, independently, in duplicate) and any processes for obtaining and confirming data from investigators.	Online Supplements
Data items	11	List and define all variables for which data were sought (e.g., PICOS, funding sources) and any assumptions and simplifications made.	Online Supplements
Risk of bias in individual studies	12	Describe methods used for assessing risk of bias of individual studies (including specification of whether this was done at the study or outcome level), and how this information is to be used in any data synthesis.	n/a
Summary measures	13	State the principal summary measures (e.g., risk ratio, difference in means).	Online

For peer review only - http://bmjopen.bmj.com/site/about/guidelines.xhtml

			Supplements
Synthesis of results	14	Describe the methods of handling data and combining results of studies, if done, including measures of consistency (e.g., I^2) for each meta-analysis.	Online Supplements
Section/topic	#	Checklist item	Reported on page #
Risk of bias across studies	15	Specify any assessment of risk of bias that may affect the cumulative evidence (e.g., publication bias, selective reporting within studies).	n/a
Additional analyses	16	Describe methods of additional analyses (e.g., sensitivity or subgroup analyses, meta-regression), if done, indicating which were pre-specified.	n/a
**RESULTS**			
Study selection	17	Give numbers of studies screened, assessed for eligibility, and included in the review, with reasons for exclusions at each stage, ideally with a flow diagram.	Online Supplements
Study characteristics	18	For each study, present characteristics for which data were extracted (e.g., study size, PICOS, follow-up period) and provide the citations.	Online Supplements
Risk of bias within studies	19	Present data on risk of bias of each study and, if available, any outcome level assessment (see item 12).	n/a
Results of individual studies	20	For all outcomes considered (benefits or harms), present, for each study: (a) simple summary data for each intervention group (b) effect estimates and confidence intervals, ideally with a forest plot.	Online Supplements
Synthesis of results	21	Present results of each meta-analysis done, including confidence intervals and measures of consistency.	14
Risk of bias across studies	22	Present results of any assessment of risk of bias across studies (see Item 15).	n/a
Additional analysis	23	Give results of additional analyses, if done (e.g., sensitivity or subgroup analyses, meta-regression [see Item 16]).	n/a
**DISCUSSION**			
Summary of evidence	24	Summarize the main findings including the strength of evidence for each main outcome; consider their relevance to key groups (e.g., healthcare providers, users, and policy makers).	14-19
Limitations	25	Discuss limitations at study and outcome level (e.g., risk of bias), and at review-level (e.g., incomplete retrieval of identified research, reporting bias).	Online Supplements
Conclusions	26	Provide a general interpretation of the results in the context of other evidence, and implications for future research.	14-19
**FUNDING**			
Funding	27	Describe sources of funding for the systematic review and other support (e.g., supply of data); role of funders for the systematic review.	20

For peer review only - http://bmjopen.bmj.com/site/about/guidelines.xhtml
